# Stimuli Responsive Silylene: Electromerism Induced Reversible Switching Between Mono‐ and Bis‐Silylene

**DOI:** 10.1002/anie.202211115

**Published:** 2022-10-21

**Authors:** Ravi Yadav, Xiaofei Sun, Ralf Köppe, Michael T. Gamer, Florian Weigend, Peter W. Roesky

**Affiliations:** ^1^ Institute of Inorganic Chemistry Karlsruhe Institute of Technology (KIT) Engesserstraße 15 76131 Karlsruhe Germany; ^2^ Fachbereich Chemie Philipps-Universität Marburg Hans-Meerwein-Strasse 4 35032 Marburg Germany

**Keywords:** Electromerism, Low-Valent Silicon, Main Group, Silylene, Stimuli Responsive

## Abstract

Electromerism is a very well‐known phenomenon in transition metal chemistry. In main group chemistry, this concept has only started getting attention recently. We report stimuli responsive low‐valent silicon compounds exhibiting electromerism. A mixed‐valent silaiminyl‐silylene **1**, [**L**Si−Si(NDipp)**L**] (**L=**PhC(N^*t*^Bu)_2_), was synthesized in a single step from amidinate‐chlorosilylene. Compound **1** has two interconnected Si atoms in formally +I and +III oxidation states. Upon treatment with Lewis acidic Cu^I^X (X=mesityl, Cl, Br, I), electron redistribution occurs resulting in the formation of [{**L**Si(NDipp)Si(**L**)}−CuX], in which both silicon atoms are in the +II formal oxidation state. Removal of the copper center from [{**L**Si(NDipp)Si(**L**)}−CuX] by using a Lewis basic carbene led to reformation of the precursor [**L**Si−Si(NDipp)**L**]. Thus, the process is fully reversible. This showcases the first example of Lewis acid/base‐induced reversible electromerism in silicon chemistry.

## Graphical abstract

A mixed valent silicon compound is synthesized in a one‐pot reaction. The mixed valency of the compound allowed reversible electromerism under the influence of Lewis‐acid/base acting as external stimuli. A mono‐silylene ligand could be transformed to bis‐silylene by adding Cu^I^ salts. Upon removal of Cu^I^ salts, the bis‐silylene motif reverts back to mono‐silylene.

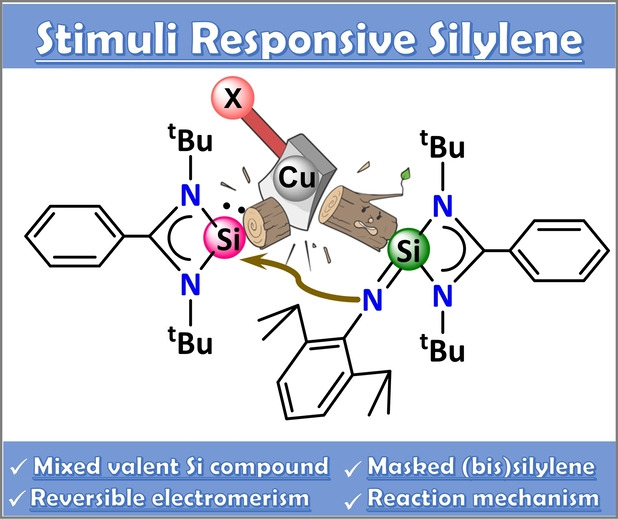

Inception from laboratory curiosity, the chemistry of silylene has crossed many milestones, such as, usage in small molecule activation, stabilization of otherwise elusive species and efficient ligands in metal‐mediated catalytic transformations of organic substrates.^[1]^ The strong σ‐donor property of silylenes has enabled their coordination complexes across the periodic table.^[2]^ Recently, one of the advancements in this area was the utilization of (bis)silylenes as bidentate chelating and strong σ‐donating ligands to obtain various coordination compounds.[^1g^, ^2e^, ^3^] Some of these (bis)silylene‐metal complexes have also been tested in catalysis and were found to be more efficient than the corresponding (bis)phosphine‐metal complexes.[^1g^, ^3a^, ^4^]

Valence tautomerism (electromerism) in metal complexes is described as electron redistribution between metal and ligand without altering the structural motif. This phenomenon is well‐known in transition metal complexes.^[5]^ A benchmark example is the cobalt bis(dioxolene)(byp) (byp=bipyridine) complex, in which the cobalt center can switch between Co^II^ and Co^III^ formal oxidation states upon shifting the temperature.^[5f]^ In contrast to transition metal complexes, stimuli‐induced electron redistribution in main group chemistry is relatively less known.^[5c]^ Moreover, electromerism is even more scarce in the case of low‐valent main group chemistry. In 1986, Arduengo reported Lewis acid/base induced electromerism in 10‐P‐3 compound, where the phosphorus atom can be reversibly switched between the P^I^ and P^III^ formal oxidation states.^[6]^ In 2007, Vaid and co‐workers have shown a remarkable example of Lewis‐base‐induced electromerism in a Ge‐porphyrin complex.^[7]^

Stimuli‐induced electromerism in low‐valent silicon chemistry is even more rare and only two reports have appeared till date. Recently, the Driess group has shown redox‐induced electromerism in the formally zero‐valent silicon species **A** by using a redox non‐innocent (bis)silylene substituted *ortho‐*carborane ligand (Figure 1a).^[8]^ When compound **A** is subjected to one electron reduction, the formally zero‐valent Si^0^ center oxidizes to Si^I^ accompanied by a two electron reduction of the *ortho*‐carborane ligand backbone (Figure 1a). Very recently, Iwamoto and co‐workers have reported a phase‐dependent electromerism in silylone **C** (Figure 1b).^[9]^ In addition, different isomers of silylated low‐valent group 14 compounds (Si, Ge, Sn) may form featuring different electronic configurations, for example, equilibrium between disilene and silylene through 1,2‐silyl shift.^[10]^

**Figure 1:**
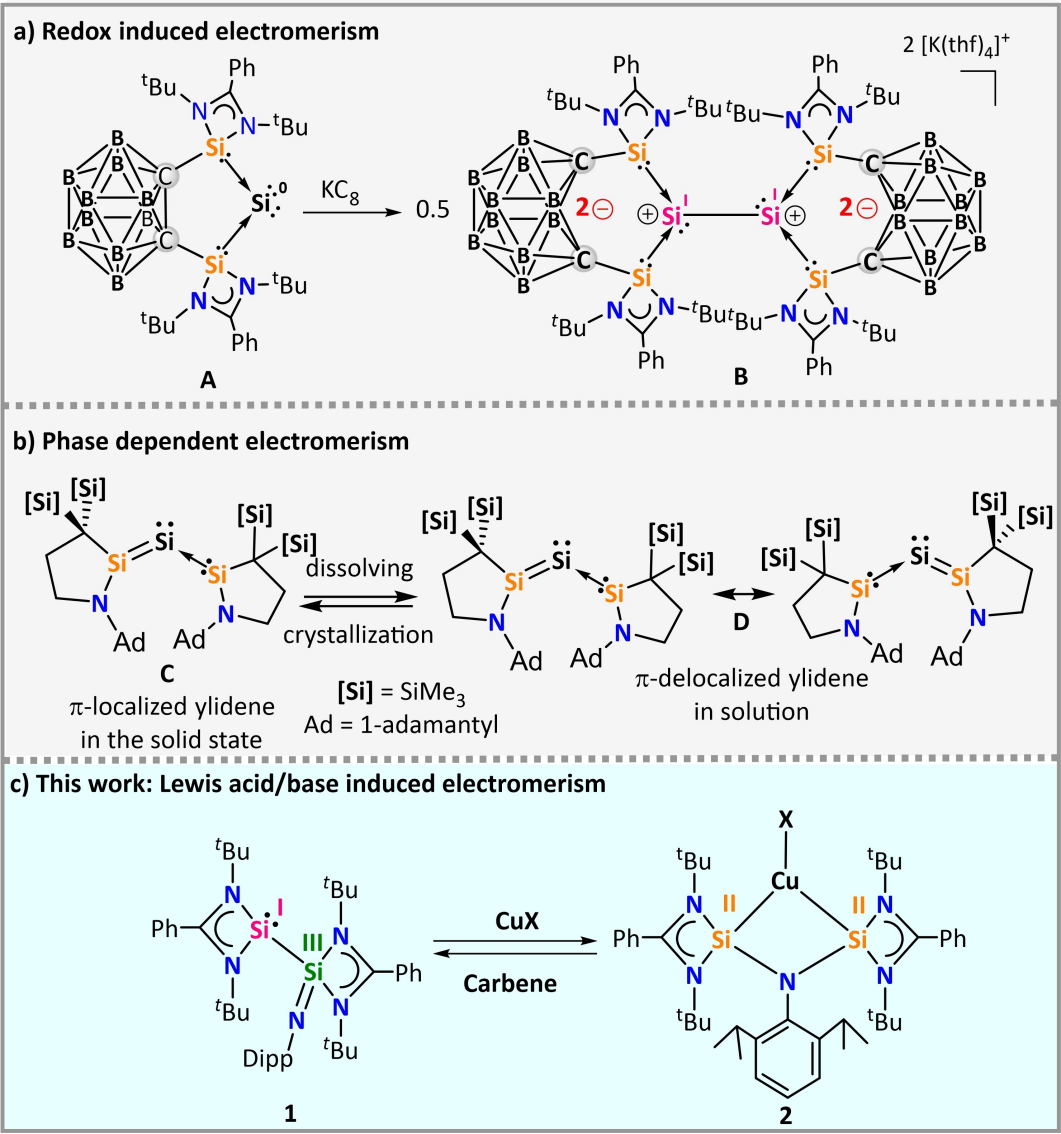
a) Redox‐induced electromerism in silylone **A**,^[8]^ b) Phase‐dependent electromerism in silylone **C**,^[9]^ c) Overview of this work.

So and co‐workers reported silaiminyl‐silylene [**L**Si−Si(NDipp)**L**] (**1**; Figure 1c) and established the molecular structure in the solid state.^[11]^ Compound **1** has two silicon atoms in different formal oxidation states of +I and +III. Upon adding external stimuli such as Lewis acid or base, compound **1** showed electromerism by switching between the mono and (bis)silylene motifs. To the best of our knowledge, this is the first example of Lewis acid/base induced reversible electromerism in silicon chemistry.

However, **1** was obtained only as a side product which could not be purified.^[11]^ Herein, we present an unusual but highly efficient one‐pot reaction for multigram‐scale synthesis of **1**. By our route, compound **1** could be isolated in 90 % yield as free flowing yellow powder by the reaction of [**L**SiCl]^[12]^ with [DippN(H)Li]^[13]^ (Dipp=2,6‐diisopropylphenyl) (Scheme 1). Compound **1** was fully characterized in solution and solid state. Its ^1^H NMR spectrum showed two resonances (*δ*=1.25 and 1.28 ppm) for 36 protons of the ^*t*^Bu‐groups and a doublet at *δ*=1.64 ppm for the methyl protons of the Dipp group. The ^29^Si{^1^H} NMR spectrum showed two resonances (*δ*=−61.7 (Si=N) and 31.8 (silylene) ppm) for the two distinct silicon centers. The ^29^Si{^1^H} NMR chemical shifts also match well with the corresponding calculated shifts at *δ*=−68 (Si=N) and 35 (silylene) ppm (see below). The formal oxidation states of Si atoms are +I and +III.

**Scheme 1:**
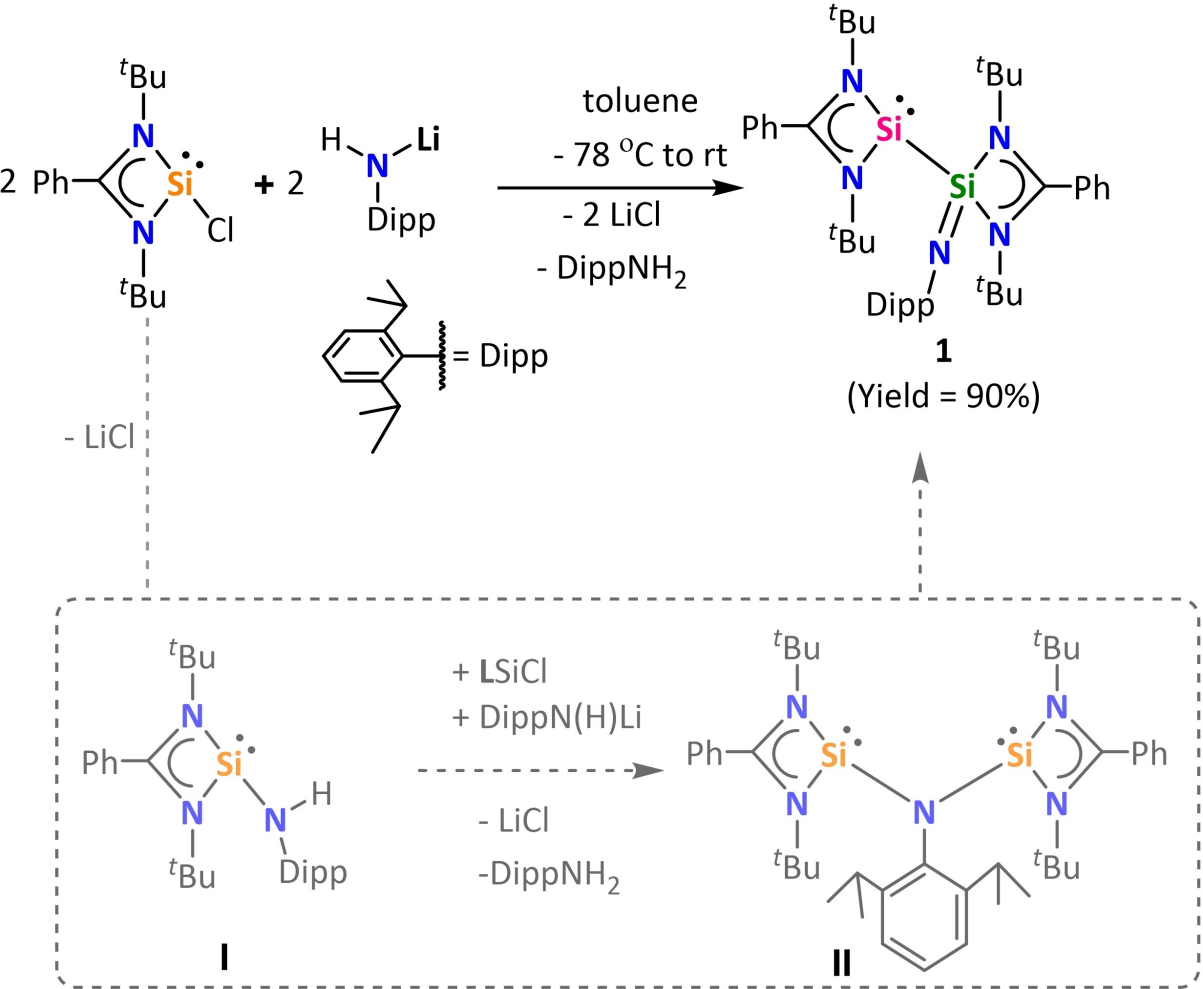
Synthesis of silaiminyl‐silylene (**1**).

The reaction of [**L**SiCl] and [DippN(H)Li] presumably first undergoes a salt metathesis reaction resulting in the amidosilylene intermediate (**I**) as shown in Scheme 1. The postulated intermediate further undergo deprotonation to give the corresponding lithium complex and DippNH_2_. The lithiated complex, upon reaction with another equivalent of [**L**SiCl] may give intermediate **II**, which rearranges to compound **1**. However, we could not trap or observe any of the possible intermediates involved thus a concerted reaction pathway towards the formation of **1** cannot be ruled out. The formation of intermediate **II** would indicate the involvement of a (bis)silylene intermediate. By density functional calculations (see below) we found that compound **1** is only 9 kJ mol^−1^ more stable than the (bis)silylene intermediate **II**. Further we were intrigued if compound **1** could isomerize to its (bis)silylene conformer **II**. Synthetically, the two forms could not be interconverted by either cooling or heating.

Interestingly, addition of mesityl‐copper to a toluene solution of **1** showed immediate color change from yellow to deep red. The ^29^Si{^1^H} NMR spectrum showed a single resonance at *δ*=−9.7 ppm indicating a symmetric species. The pentane solution of the reaction mixture furnished single crystals of compound **2 a**. The molecular structure of compound **2 a** showed the formation of (bis)silylene‐coordinated mesityl copper complex (Figure 2). Along this line, a series of different copper‐halide derivatives (**2 b**, Cl; **2 c**, Br; and **2 d**, I) were prepared in a similar manner (Figure 2). All of the compounds (**2 a**–**d**) showed similar NMR spectra so only those of **2 a** are discussed in details below. In the ^1^H NMR spectrum of **2 a**, the protons of the ^*t*^Bu‐groups show two broad resonances at *δ*=0.96 and 1.45 ppm. The Dipp‐C*H* protons feature a distinct septet at *δ*=4.03 ppm, upfield‐shifted as compared to **1** (*δ*=4.58). The Dipp‐C*H*_3_ protons are overlapped by the ^*t*^Bu protons at *δ*=1.45 ppm. In contrast to compound **1**, the ^29^Si{^1^H} NMR spectrum of **2 a** shows a single resonance at *δ*=−9.7 ppm, indicating the presence only one type of Si atom. The molecular structures of **2 a**–**d** are in agreement with the NMR spectroscopy. The average Si−Cu bond lengths in **2 a**–**d** are ranging from 2.2801(6) to 2.3099(7) Å and are in line with previously reported silylene‐Cu^I^ complexes.^[14]^ The average Si−N(Dipp) bond lengths are in the range 1.7837(13)–1.7895(2) Å, also in agreement with previously known bond lengths for Si−N bonds in related systems.[^14b^, ^14c^, ^15^] Selected bond lengths and chemical shift for ^29^Si{^1^H} NMR resonances are provided in Table 1.

**Figure 2:**
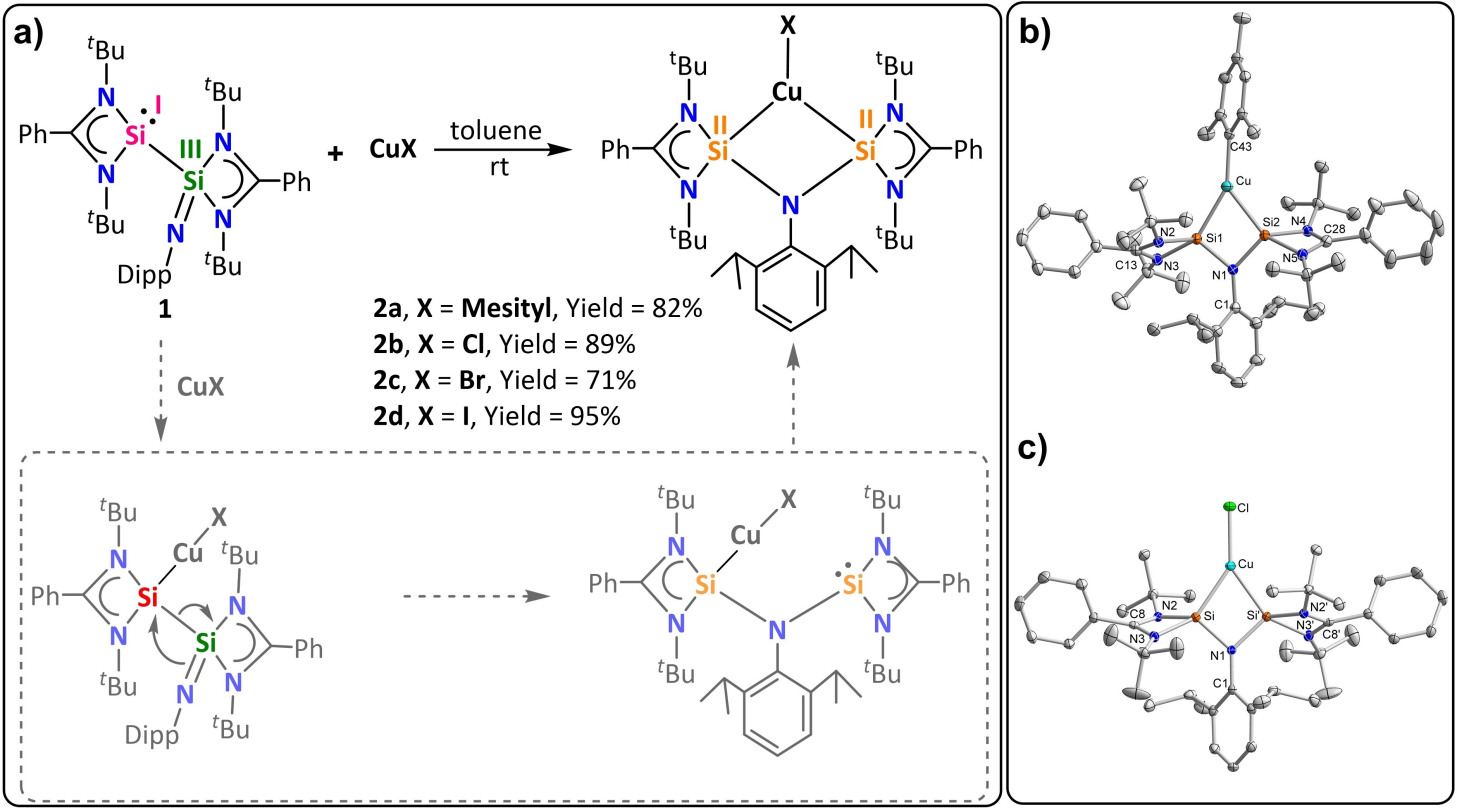
a) Synthesis of (bis)silylene‐copper complexes. b) Molecular structure in the solid state of **2 a** (thermal ellipsoids are displayed at 40 % probability, hydrogen atoms are omitted for clarity), selected bond lengths [Å] for **2 a**: Cu−Si2 2.3355(6), Cu−Si1 2.2801(6), Cu−C43 1.961(2), Si2−N4 1.8950(2), Si2−N1 1.7930(2), Si1−N1 1.7850(2), Si2−N5 1.8420(2), Si1−N2 1.8880(2), Si1−N3 1.840(2), Si⋅⋅⋅Si 2.6149(8). c) Molecular structure in the solid state of **2 b** (thermal ellipsoids are displayed at 40 % probability, hydrogen atoms are omitted for clarity), selected bond lengths [Å] for **2 b**: Cu−Cl 2.1797(6), Cu−Si′ 2.2847(5), Cu−Si 2.2846(5), Si−N1 1.7837(13), Si−N2 1.8750(13), Si−N3 1.8193(13), N1−C1 1.449(3), N2−C8 1.3460(2), N3−C8 1.3420(2), N3−C19 1.4760(2), Si⋅⋅⋅Si′ 2.6187(8).

**Table 1:** Selected bond lengths and *δ* ^29^Si{^1^H} for **2**.

Compound	Si−Cu [Å]	Si−N(Dipp) [Å]	^29^Si{^1^H} [ppm]
**2 a** (Si−Cu−Mes)	2.2801(6) 2.3355(6)	1.7895(2) 1.7930(2)	−9.7
**2 b** (Si−Cu−Cl)	2.2846(5)	1.7837(13)	−8.9
**2 c** (Si−Cu−Br)	2.2762(7) 2.3099(7)	1.7870(2), 1.7840(2)	−6.6
**2 d** (Si−Cu−I)	2.2887(5)	1.7863(12),	−5.9

The (bis)silylene ligand in **2** is the first silylene analogue of the widely used diphosphinoamine ligands (PNP).^[16]^ The copper‐coordinated (bis)silylene ligand is formed upon electromerisation of silaiminyl‐silylene (**1**). As indicated in Figure 2 (dashed box), the electromerisation is induced by coordination of the lone pair on one of the Si atoms in **1**. Then the coordinatively unsaturated Cu center compels the electron redistribution to generate one more silylene moiety, which subsequently coordinates to the Cu atom to form **2** (Figure 2). The formal oxidation states of the Si atoms in **1** are +I (silylene center) and +III (Si=N center) and upon coordination to Cu^I^ the oxidation states redistribute to +II for each Si center in **2**. This is the first example of Lewis acid‐induced electromerism in silicon chemistry.

We were intrigued whether this electromerisation process is reversible, or in other words, whether the (bis)silylene framework could revert to compound **1** upon removal of the Cu center. An NMR scale reaction between **2 d** and ITMe^[17]^ (1,3,4,5‐tetramethylimidazol‐2‐ylidene) in C_6_D_6_ resulted in the formation of compound **1** and an insoluble compound, which may correspond to the complex [ITMe−CuI] (Scheme 2, see Section 2 of Supporting Information). This shows the stimuli responsive nature of silaiminyl‐silylene, which can switch back and forth between mono and bis‐silylene motifs. Such reversible electromerism of silaiminyl‐silylenes can be fascinating for metal ligand cooperativity for different bond activation reactivities and during catalytic cycles.

**Scheme 2:**
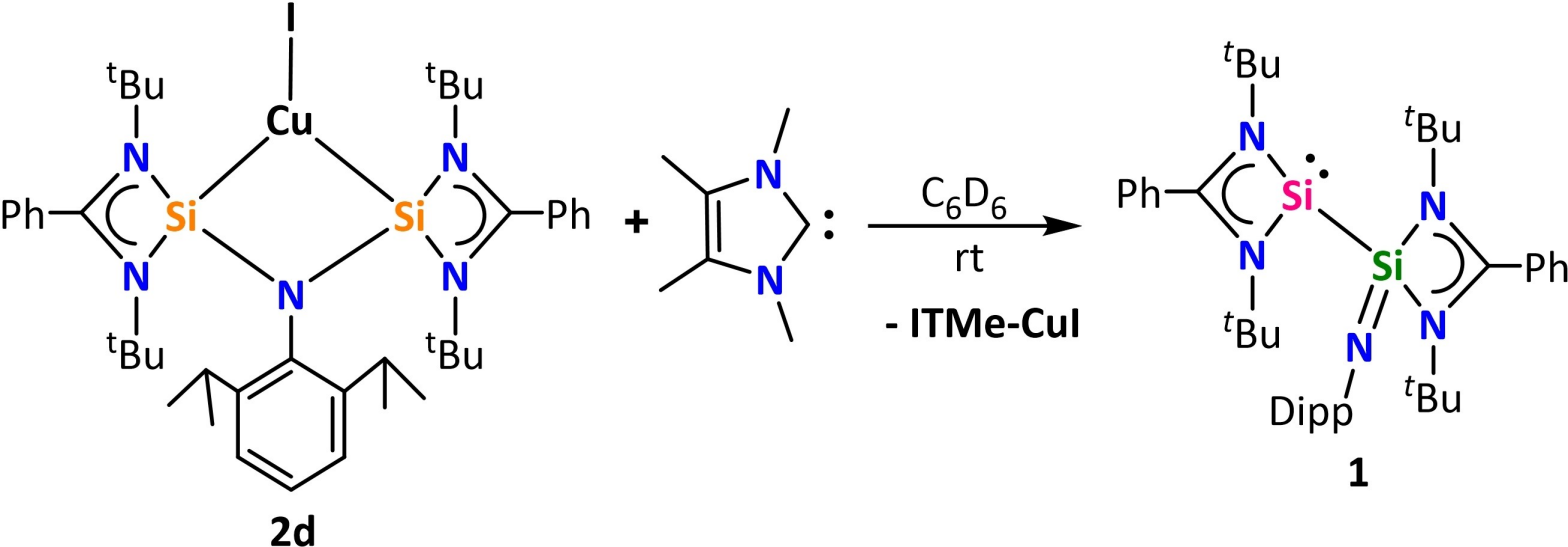
Lewis base‐induced transformation of (bis)silylene to silaiminyl‐silylene.

To improve the knowledge on the bonding situation in the reactant **1** as well as in the product molecules, quantum chemical DFT calculations (functional BP‐86, SVP basis sets) were carried out.^[18]^ First, we were interested in the energetic relationship between the silaiminyl‐silylene, **1**, used and its (bis)silylene isomer, II. According to our calculations, **1** is favored by only about 9 kJ mol^−1^, so that—according to the van't Hoff equation—about 3 % of the ligand should be present as (bis)silylene (**II**) in solution at room temperature. It is noted in passing that solvent effects (treated via COSMO^[19]^ with *ϵ*=2.4 for toluene) slightly increase the preference for **1** to 16 kJ mol^−1^. Ahlrichs‐Heinzmann population analyses^[20]^ support the silicon centers to be in the formal oxidation states +I and +III in **1** as well as +II in the theoretical isomer **II** (partial charges Q: **1**: −0.07, +0.38; **II**: +0.15).

By careful optimization of the reaction pathway, an energetic barrier between **II** and **1** of 59 kJ mol^−1^ (65 kJ mol^−1^ in solution) is found; similar numbers are found with a hybrid functional and more flexible basis sets or when dispersive interactions are taken into account; enthalpy contributions to the barrier at room temperature are below 1 kJ mol^−1^; for details see Supporting Information (and movie in the Supporting Information). Therefore, a thermodynamic equilibrium between both two isomers can be safely assumed at room temperature. The calculated ^29^Si NMR shifts^[21]^ of **1** (*δ*(calc.)=−68, 35 ppm) confirm the experimental findings (*δ*(exp.)=−61.7, 31.8 ppm). The calculated shift for Si atoms of the (bis)silylene **II** was expected to be close to −6.5 ppm, however, experimentally it could not be detected due to its expected low concentration in the equilibrium. Moreover, there are no signals (for either the ^*t*^Bu‐group or the isopropyl‐protons of the Dipp‐group) corresponding to any other species (conformer **II**) in the ^1^H NMR spectrum of **1**. In order to determine the reason for the preferred realization of the silaiminyl‐silylene, we have replaced the Dipp residue connected to the nitrogen atoms with the less bulky phenyl group on both ligand isomers in further calculations. In this situation the (bis)silylene [**L**Si−N(Ph)−Si**L**] (**L**=PhC(N^*t*^Bu)_2_) is preferred by about 53 kJ mol^−1^ compared to the silaiminyl‐silylene [**L**Si−Si(NPh)**L**]. This is in accordance when comparing the calculated distances of the bonds to the central nitrogen atom of both (bis)silylenes. The Si−N and N−C bonds are about 1.5 to 2 pm shorter in [**L**Si−N(Ph)−Si**L**] (*r*(Si−N) 1.819, r(C−N) 1.417 Å) compared to [**L**Si−N(Dipp)−Si**L**] (*r*(Si−N) 1.841, *r*(C−N) 1.431 Å). The formally nonbonding Si⋅⋅⋅Si distance in theoretical **II** of 2.647 Å is in the range of a Si−Si single bond in hexa‐*tert*‐butyldisilane—the molecule with the longest Si−Si bond (2.511 Å).^[22]^ The corresponding value in ([**L**Si−N(Ph)−Si**L**]) is calculated to be 2.915 Å, but both are of no energetic importance. While preparing this manuscript, the Driess group reported the isolation of [**L**Si−N(Ph)−Si**L**] in 65 % yield.^[23]^ In accordance with our calculations [**L**Si−N(Ph)−Si**L**] exists exclusively as the (bis)silylene isomer in solution and solid state.

Further evidence is provided by the absence of a bond‐critical point between the silicon centers in the electronic charge density plot (see Figure 3) obtained after a QTAIM analysis.^[24]^ Furthermore, local stretching force constants assessing the bond strength were determined by means of the plugin LModeA‐nano^[25]^ of the visualization program pymol. The Si⋅⋅⋅Si local mode force constants in both [**L**Si−N(Dipp)−Si**L**] and [**L**Si−N(Ph)−Si**L**] is calculated to be about 0.5 mdyn Å^−1^ despite the huge difference in their Si⋅⋅⋅Si bond lengths. The independence is best interpreted as a “local deformation force constant” of the angle <(Si−N−Si) but not due to a bonding interaction between both silicon centers.

**Figure 3:**
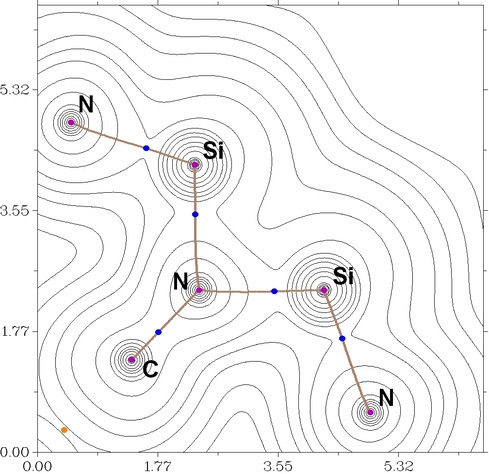
Electronic density plot, bond paths and bond critical points (in blue) of the bis silylene **II**.

To investigate the bonding situation of the complexes, we have examined the reaction product **2 d** with CuI as an example. For the complex formation, a heat of reaction of −292 kJ mol^−1^ is calculated. This huge value is in good agreement with other metal complexes involving subvalent silicon species as well as in carbonyl compounds. Thus, on one hand, the bonding of molecular SiO to palladium atoms in [PdSiO], for example, is determined to be −182 kJ mol^−1^,^[26]^ on the other hand, the complex bonding of CuCl to CO is determined to be −155.2 kJ mol^−1^.^[27]^ The reaction of complex **2 d** with the carbene, ITMe, leads to a reaction energy of −6 kJ mol^−1^ (theoretical reaction in the gas phase), caused by the strong bond after formation of [ITMe−CuI]. Furthermore, its low solubility supports this experimental behavior. The rearrangement of the ligand from **II** to **1** that accompanies this reaction is in line with the theoretical findings.

In summary, we have established an efficient one‐pot synthesis of the mixed‐valent silylene compound **1**. Compound **1** has two silicon atoms in different oxidation states (+I and +III). It showed interesting property of stimuli‐induced electromerism. Upon reacting compound **1** with Lewis acids, CuX (X=Cl, Br, I, Mes), electron redistribution in the (Si−Si=N) motif occurs to produce a series of bis(silylene) Cu complexes **2 a**–**d**, in which both silicon atoms are in the +II oxidation state. The (bis)silylene motif can be transformed back to the (mono)silylene motif by removing CuX from compound **2** using a strong Lewis base. The electromerism of the ligand is supported by theoretical calculations. The reason for the chemical equilibrium is explained by the sterically demanding Dipp group, so that the formation of (bis)silylenes by variation of this group is rather likely. The complexation behavior of the ligand to CuI is confirmed by the calculations as well. This is the first observation of Lewis acid/base‐induced reversible electromerism in silicon chemistry. On demand switching between mono and (bis)silylene can possibly pave the way for hemilabile silylene ligands. This works also opens new avenues to pursue the electromerism in low valent main group chemistry.

## Conflict of interest

The authors declare no conflict of interest.

## Supporting information

As a service to our authors and readers, this journal provides supporting information supplied by the authors. Such materials are peer reviewed and may be re‐organized for online delivery, but are not copy‐edited or typeset. Technical support issues arising from supporting information (other than missing files) should be addressed to the authors.

Supporting Information

Supporting Information

Supporting Information

Supporting Information

Supporting Information

Supporting Information

## Data Availability

The data that support the findings of this study are available in the Supporting Information of this article.
