# Supplementary material for: Stimuli Responsive Silylene: Electromerism Induced Reversible Switching Between Mono‐ and Bis‐Silylene
Source: Angew Chem Int Ed Engl. 2022 Oct 21;61(47):e202211115. doi: 10.1002/anie.202211115 (PMC9828679; doi:10.1002/anie.202211115)
Supplement: Supplementary file 5 — Supporting Information [file ANIE-61-0-s006.pdf]

Angewandte Chemie
Eine Zeitschrift der Gesellschaft Deutscher Chemiker

Supporting Information

Stimuli Responsive Silylene: Electromerism Induced Reversible Switching Between Mono- and Bis-Silylene

*R. Yadav, X. Sun, R. Köppe, M. T. Gamer, F. Weigend, P. W. Roesky**

Supporting Information

Stimuli Responsive Silylene: Electromerism Induced Reversible Switching Between Mono- and Bis-Silylene.

Ravi Yadav[a], Xiaofei Sun[a], Ralf Köppe[a], Michael T. Gamer[a], Florian Weigend[b], and Peter W. Roesky[a]*

[a]*Institute of Inorganic Chemistry, Karlsruhe Institute of Technology (KIT), Engesserstraße 15, 76131 Karlsruhe (Germany). Email: roesky@kit.edu.*

[b]*Fachbereich Chemie, Philipps-Universität Marburg, Hans-Meerwein-Strasse 4, D-35032, Germany E-Mail: florian.weigend@chemie.uni-marburg.de*

Contents

1. Experimental Section S2

1.1. General Methods S2

Synthesis of [{LSi-Si(L)=NDipp}] (**1**) S3

Synthesis of [{LSi(NDipp)Si(L)}-CuMes] (**2a**) S3

Synthesis of [{LSi(NDipp)Si(L)}-CuX] (X = Cl, **2b**; X = I, **2d**) S4

Synthesis of [{LSi(NDipp)Si(L)}-CuBr] (**2c**) S5

2. NMR Spectra of compounds **1**-**3** S7

3. NMR experiments for conversion of **2d** to **1** upon adding ITMe (1,3,4,5-tetramethylimidazol-2-ylidene) S24

4. Single crystal X-ray diffraction analysis S26

4.1. Table S1 Crystal data and structure refinement S27

4.2. Crystal Structures S28

5. Quantum chemical calculations S32

6. References S57

1. Experimental Section

1.1. General Methods

All the manipulations of air- and water-sensitive reactions were performed with rigorous exclusion of oxygen and moisture in flame-dried Schlenk-type glassware either on a dual manifold Schlenk line, interfaced to a high vacuum (10^{-3} torr) line or in an argon-filled MBraun glove box. Solvents were dried by using an MBraun solvent purification system (SPS 800), degassed and stored *in vacuo* over $LiAlH_4$. Elemental analyses were carried out with an Elementar vario Micro cube. Thf-d_8 and C_6D_6 were stored over Na/K alloy and were degassed by freeze-pump-thaw cycles. IR spectra were obtained on a Bruker Tensor 37 spectrometer equipped with a room temperature DLaTGS detector and a diamond ATR (attenuated total reflection) unit. ^{1}H, $^{13}C\{^{1}H\}$, and $^{29}Si\{^{1}H\}$ IG (inverse gated) NMR spectra were recorded on a Bruker Avance 400 (^{1}H: 400.30 MHz, ^{13}C: 100.67 MHz, ^{29}Si: 79.5 MHz). The chemical shifts are reported in ppm relative to external TMS (^{1}H, ^{13}C, ^{29}Si). All NMR spectra were measured at 298 K, unless otherwise specified. The multiplicity of the signals is indicated as s = singlet, d = doublet, t = triplet, sept = septet, m = multiplet and br = broad. Synthesis of [**L**SiCl][1] (**L** = PhC(N*t*Bu)$_2$), [DippN(H)Li][2] (Dipp = 2,6-diisoproplyphenyl), and mesityl-copper[3] were synthesized according to the literature procedure.

Synthesis of [{LSi-Si(L)=NDipp}] (1)

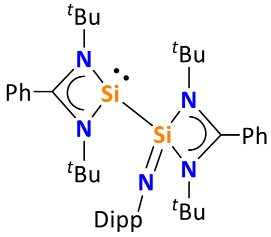

1

To a mixture of [**L**SiCl] (5 g, 16.96 mmol) and [DippN(H)Li] (3.11 g, 16.96 mmol) ice-cold 100 mL of toluene was added slowly while stirring. The reaction mixture was stirred at room temperature for 18 h. The reaction mixture was filtered through P4 frit and the residue was washed with 20 mL toluene. All the volatiles were removed from the filtrate. The product was washed 20*3 mL pentane and dried *in vacuo* to obtain a yellow solid.

Yield: 5.03 g (15.27 mmol; 90%). Anal Calcd. for ($C_{42}H_{63}N_5Si_2$): C, 72.67 ; H, 9.15 ; N, 10.09. Found: C, 72.58; H, 8.72; N, 9.93.

^{1}H NMR (400 MHz, 298 K, C_6D_6): δ [ppm] = 1.25 (s, 18H, C(CH_3)$_3$), 1.28 (s, 18H, C(CH_3)$_3$), 1.64 (d, Dipp-CH_3, 12H, $^3J_{HH}$ = 6.8 Hz), 4.58 (sept, 2H, Dipp-C*H*, $^3J_{HH}$ = 6.8 Hz), 6.92-6.96 (m, 4H, Ar-*H*), 7.0-7.08 (m, 4H, Ar-*H*), 7.24 (m, 1H, Ar-*H*), 7.33 (m, 1H, Ar-*H*), 7.38-7.43 (m, 3H, Ar-*H*).

^{13}C{^{1}H} NMR (100 MHz, 298 K, C_6D_6): δ [ppm] = 25.2 (Dipp-*C*H$_3$), 27.8 (Dipp-*C*H), 32.0 (C(*C*H$_3$)$_3$), 32.1 (C(*C*H$_3$)$_3$), 53.5 (*C*(CH$_3$)$_3$), 54.0 (*C*(CH$_3$)$_3$), 114.7, 122.6, 129.19, 129.22, 129.7, 130.1, 130.5, 132.4, 135.2, 139.4, 148.3, 153.0 (Dipp-Ar and Ph-*C*), 173.0 (N*C*N) only one signal could be seen for NCN.

^{29}Si{^{1}H} NMR (79.5 MHz, 298 K, C_6D_6): δ [ppm] = -61.7 and 31.8.

Synthesis of [{LSi(NDipp)Si(L)}-CuMes] (2a)

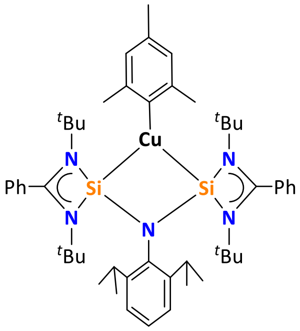

2a

Toluene (10 mL) was added to a mixture of **1** (500 mg, 0.72 mmol) and mesityl-copper (131 mg, 0.72 mmol) at room temperature. The reaction mixture immediately turned dark red. The reaction was further stirred for 3 hours at room temperature and then all the volatiles were removed. The red solid was dissolved in minimum amount of pentane and stored at -30 ^{o}C. After few days dark red-coloured crystals were obtained which were suitable for x-ray diffraction studies. The mother-liquor was decanted off and the product was dried *in vacuo*.

Yield: 520 mg (0.59 mmol; 82%). Anal Calcd. for ($C_{51}H_{74}N_5Si_2Cu$) : C, 69.85; H, 8.51; N, 7.99. Found: C, 69.54; H, 8.48; N, 7.86.

^{1}H NMR (400 MHz, 298 K, C_6D_6): δ [ppm] = 0.96 (br, $\Delta\nu_{1/2}$ ≈ 28 Hz, 18H, C(C*H*$_3$)$_3$), 1.45 (br, $\Delta\nu_{1/2}$ ≈ 28 Hz, 18H C(C*H*$_3$)$_3$ and 12H Dipp-C*H*$_3$), 2.51 (s, 3H, Mes-*p*-C*H*$_3$), 3.32 (s, 6H, Mes-*o*-C*H*$_3$), 4.03 (sept, 2H, Dipp-C*H*, $^3J_{HH}$ = 6.8 Hz), 6.83-6.92 (m, 6H, Ar-*H*), 7.08-7.15 (m, 3H, Ar-*H*), 7.23 (s, 1H, Mes-*H*), 7.25 (s, 1H, Mes-*H*), 7.29-7.32 (m, 4H, Ar-*H*).

^{13}C{^{1}H} NMR (100 MHz, 298 K, C_6D_6): δ [ppm] = 21.9 (Mes-*p*-CH$_3$), 24.9 (br, Dipp-*C*H$_3$), 26.8 (Dipp-*C*H), 31.0 (Mes-*o*-*C*H$_3$), 32.2 (br, C(*C*H$_3$)$_3$), 33.1 (br, C(*C*H$_3$)$_3$), 53.7 (br, *C*(CH$_3$)$_3$), 55.2 (br, *C*(CH$_3$)$_3$), 123.1, 124.8, 124.9, 129.2, 129.5, 129.8, 132.2, 133.2, 143.3, 144.9, 145.6, 160.8 (Mes-*C*, Dipp-*C*, Ph-*C*), 170.7 (N*C*N).

^{29}Si{^{1}H} NMR (79.5 MHz, 298 K, C_6D_6): δ [ppm] = -9.7.

Synthesis of [{LSi(NDipp)Si(L)}-CuX] (X = Cl, 2b; X = I, 2d)

To a mixture of **1** (500 mg, 0.72 mmol) and CuX (X = Cl, 71 mg, 0.72 mmol; X = I, 137 mg, 0.72 mmol) 10 mL of toluene was added, and the suspension was stirred for 18 hours at room temperature. Then the reaction mixture was heated to obtain a clear red solution. The solution was allowed to stand at room temperature to obtain red-coloured crystals suitable for x-ray diffraction analyses. The mother-liquor was decanted off and the product was dried *in-vacuo*.

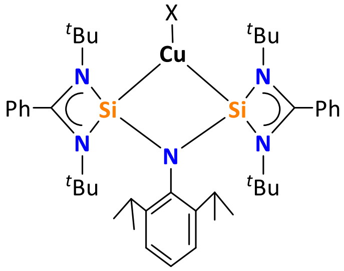

2b; X = Cl
2d; X = I

Analytics for **2b**: Yield: 510 mg (0.642 mmol; 89%). Anal Calcd. for **2b**·(toluene) ($C_{42}H_{63}N_5Si_2CuCl\cdot(C_7H_8)$) : C, 66.48; H, 8.08; N, 7.91. Found: C, 66.82; H, 7.44; N, 6.98.

^{1}H NMR (400 MHz, 298 K, d_8-thf): δ [ppm] = 0.86 (br, $\Delta\nu_{1/2}$ ≈ 28 Hz, 18H, C(C*H*$_3$)$_3$), 1.26-1.49 (br, $\Delta\nu_{1/2}$ ≈ 108 Hz, 18H C(C*H*$_3$)$_3$ and 12H Dipp-C*H*$_3$), 3.78 (sept, 2H, Dipp-C*H*, $^3J_{HH}$ = 6.5 Hz), 6.93 (t, 1H, Dipp-p-*H*, $^3J_{HH}$ = 7.6 Hz), 7.12 (d, 2H, Dipp-m-*H*, $^3J_{HH}$ = 7.6 Hz), 7.11-7.50 (m, 10H, Ph-*H*).

^{13}C{^{1}H} NMR (100 MHz, 298 K, d_8-thf): δ [ppm] = 27.2 (Dipp-*C*H), 30.7 (Dipp-*C*H$_3$), 32.2 (br, C(*C*H$_3$)$_3$), 33.0 (br, C(*C*H$_3$)$_3$), 54.5 (br, *C*(CH$_3$)$_3$), 55.7 (br, *C*(CH$_3$)$_3$), 123.6, 125.1, 128.5, 128.7, 129.5, 130.3, 130.8, 133.2, 143.9, 144.9 (Dipp-Ar and Ph-*C*), 163.0 (N*C*N). One signal for Dipp-C*H*$_3$ is covered by d_8-thf signal at 25.1 ppm.

^{29}Si{^{1}H} NMR (79.5 MHz, 298 K, d_8-thf): δ [ppm] = -8.9.

Analytics for **2d**: Yield of **2d** = 610 mg (0.69 mmol; 95%). Anal Calcd. for **2d** ($C_{42}H_{63}N_5Si_2CuI$) : C, 57.03; H, 7.18; N, 7.92. Found: C, 56.19; H, 6.53; N, 7.79.

^{1}H NMR (400 MHz, 298 K, d_8-thf): δ [ppm] = 0.90 (br, $\Delta\nu_{1/2}$ ≈ 70 Hz, 18H, C(C*H*$_3$)$_3$), 1.33 (br, $\Delta\nu_{1/2}$ ≈ 80 Hz, 18H C(C*H*$_3$)$_3$ and 12H Dipp-C*H*$_3$), 3.78 (sept, 2H, Dipp-C*H*, $^3J_{HH}$ = 6.8 Hz), 6.96 (t, 1H, Dipp-p-*H*, $^3J_{HH}$ = 7.6 Hz), 7.14 (d, 2H, Dipp-m-*H*, $^3J_{HH}$ = 7.6 Hz), 7.42-7.52 (m, 10H, Ph-*H*).

^{13}C{^{1}H} NMR (100 MHz, 298 K, d_8-thf): δ [ppm] = 27.3 (Dipp-*C*H), 30.8 (Dipp-*C*H$_3$), 32.6 (br, C(*C*H$_3$)$_3$), 55.5 (br, *C*(CH$_3$)$_3$), 123.8, 125.3, 128.5, 128.7, 129.4, 130.5, 130.9, 133.0, 143.8, 144.8, (Dipp-Ar and Ph-*C*), 163.5 (N*C*N). One signal for Dipp-C*H*$_3$ is covered by d_8-thf signal at 25.1 ppm.

^{29}Si{^{1}H} NMR (79.5 MHz, 298 K, d_8-thf): δ [ppm] = -5.9.

Synthesis of [{LSi(NDipp)Si(L)}-CuBr] (2c)

To a mixture of **1** (500 mg, 0.72 mmol) and CuBr (103 mg, 0.72 mmol) 10 mL of thf was added, and stirred for 18 hours at room temperature. The reaction mixture was concentrated till incipient crystallization and then stored at -30 ^oC. After few days red coloured crystals were collected by decanting off the mother-liquor and subsequently dried *in-vacuo*.

Yield: 430 mg (0.530 mmol; 71%). Anal Calcd. for **2c** ($C_{42}H_{63}N_5Si_2CuBr$): C, 60.23; H, 7.58; N, 8.36. Found: C, 61.16; H, 7.47; N, 8.78.

^{1}H NMR (400 MHz, 298 K, d_8-thf): δ [ppm] = 0.89 (br, $\Delta\nu_{1/2}$ ≈ 35 Hz, 18H, C(C*H*$_3$)$_3$), 1.32 (br, $\Delta\nu_{1/2}$ ≈ 125 Hz, 18H C(C*H*$_3$)$_3$ and 12H Dipp-C*H*$_3$), 3.78 (sept, 2H, Dipp-C*H*, $^3J_{HH}$ = 6.8 Hz), 6.95 (t, 1H, Dipp-p-*H*, $^3J_{HH}$ = 7.6 Hz), 7.13 (d, 1H, Dipp-m-*H*, $^3J_{HH}$ = 7.6 Hz), 7.42-7.50 (m, 10H, Ph-*H*).

^{13}C{^{1}H} NMR (100 MHz, 298 K, d_8-thf): δ [ppm] = 27.3 (Dipp-*C*H), 30.8 (Dipp-*C*H$_3$), 32.0 (br, C(*C*H$_3$)$_3$), 33.0 (br, C(*C*H$_3$)$_3$), 55.8 (br, *C*(CH$_3$)$_3$), 123.7, 125.2, 128.5, 128.7, 129.4, 130.4, 130.8,

133.1, 143.9, 144.9 (Dipp-Ar and Ph-*C*), 163.5 (N*C*N). One signal for Dipp-CH_3 is covered by d_8-thf signal at 25.1 ppm.

^{29}Si{^{1}H} NMR (79.5 MHz, 298 K, d_8-thf): δ [ppm] = -6.6.

2. NMR Spectra of compounds 1-3

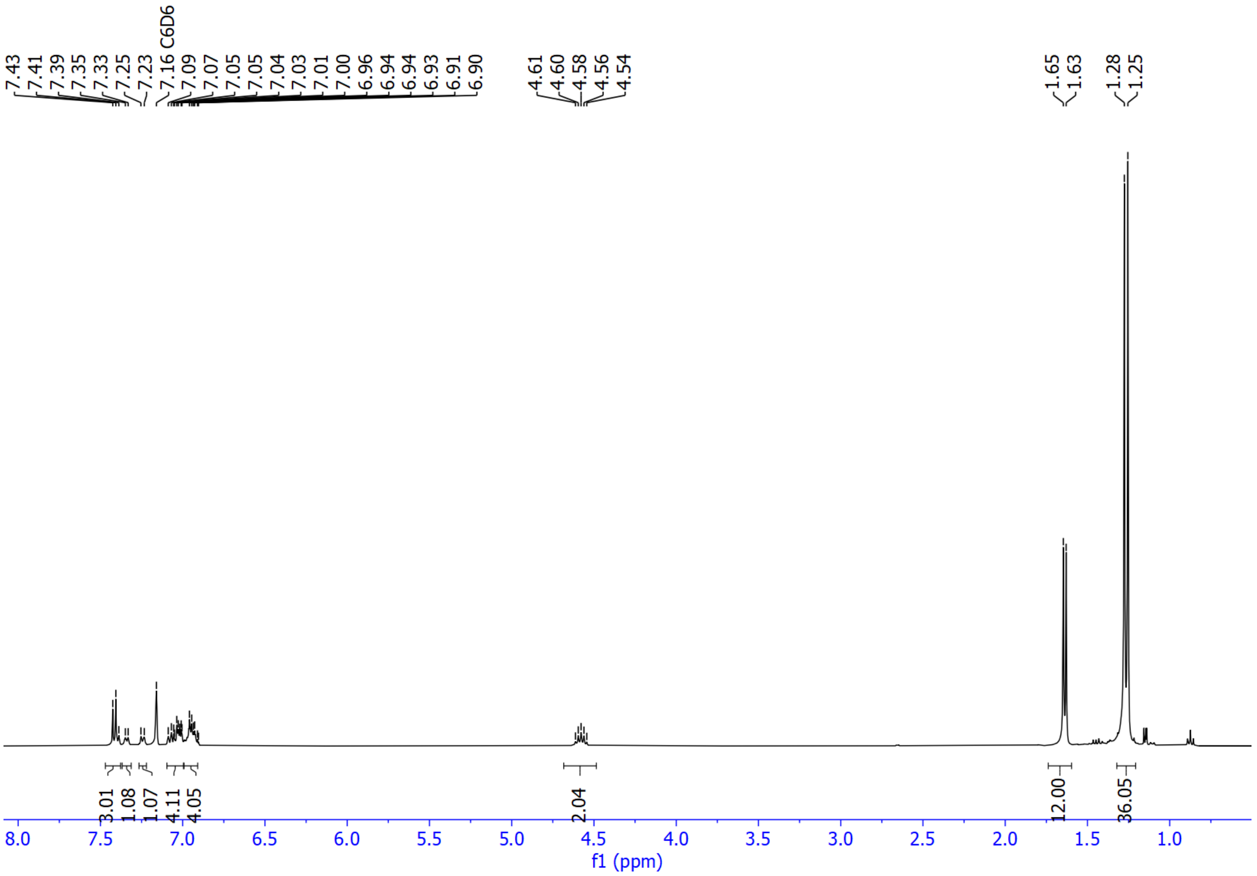

Figure S1. ^{1}H NMR (400 MHz, 298 K, C_6D_6) spectrum of compound **1**.

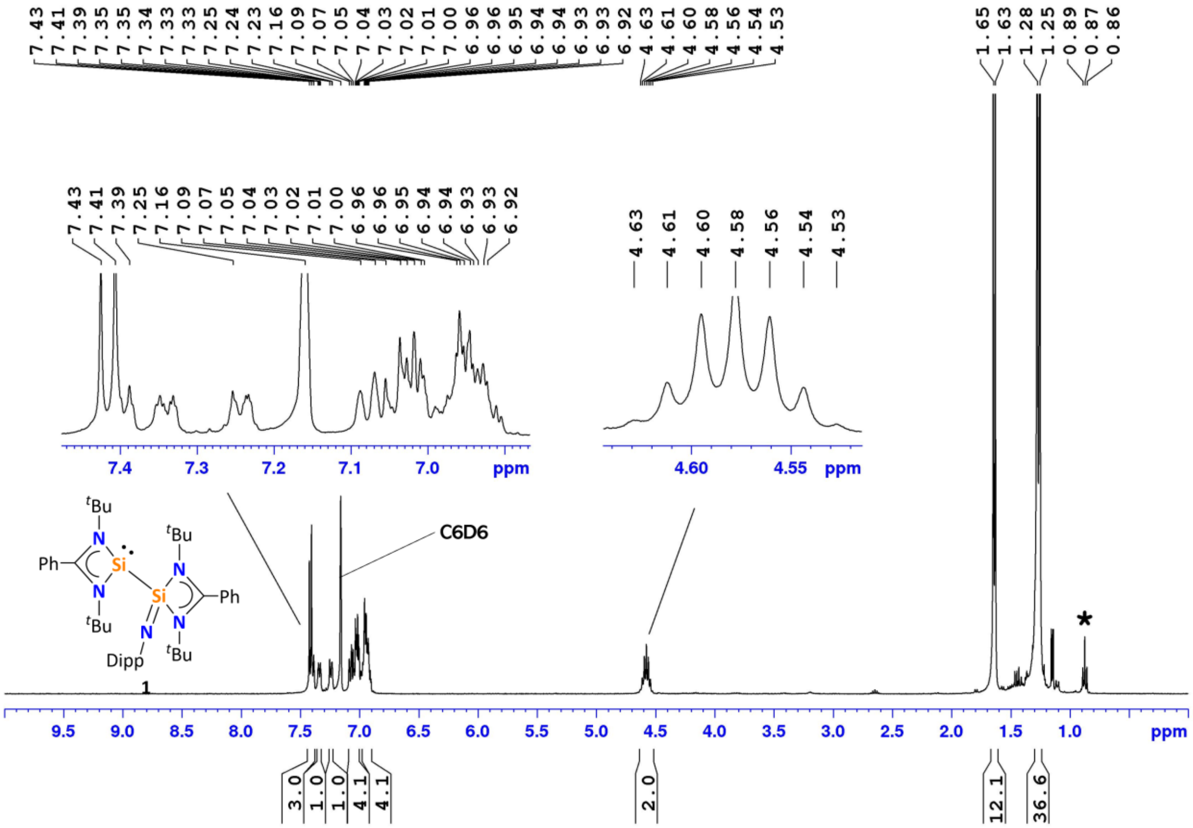

Figure S2. Zoomed-in ^{1}H NMR (400 MHz, 298 K, C_6D_6) spectrum of compound **1**. * = residual pentane.

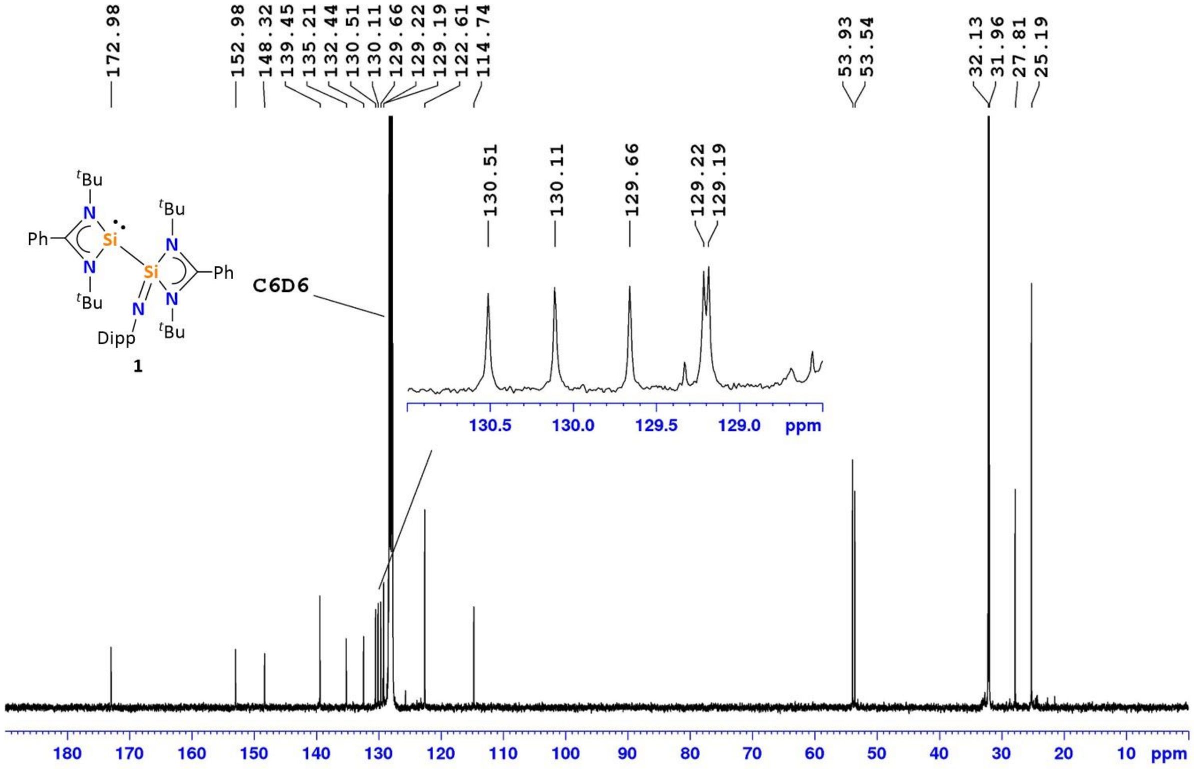

Figure S3. $^{13}C\{^1H\}$ NMR (100 MHz, 298 K, C_6D_6) spectrum of compound **1**.

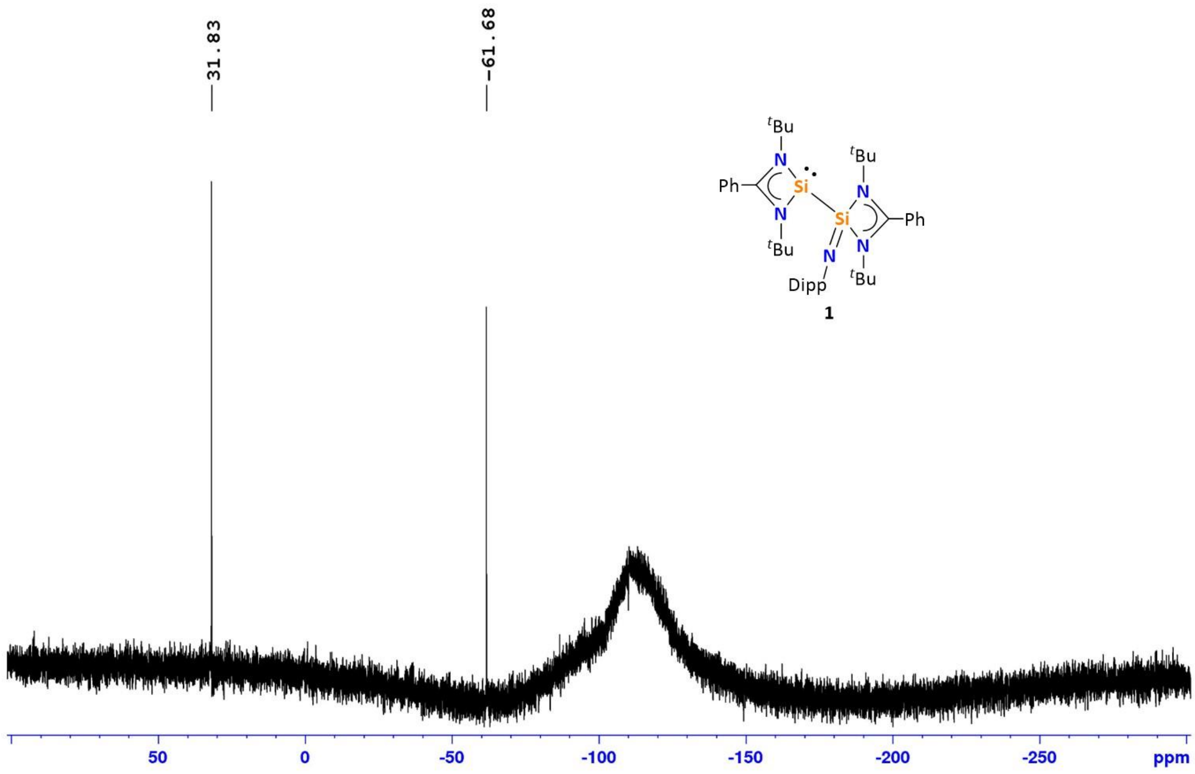

Figure S4. $^{29}Si\{^1H\}$ NMR (79.5 MHz, 298 K, C_6D_6) spectrum of compound **1**.

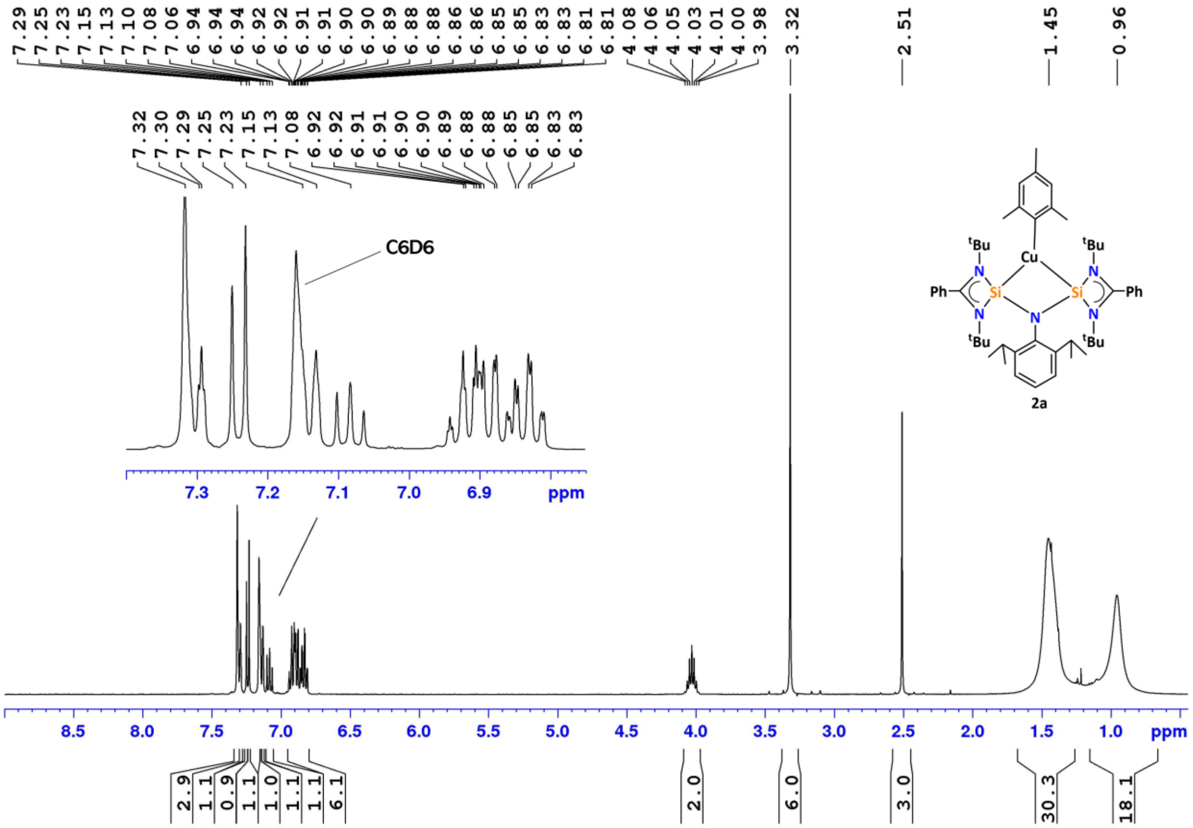

Figure S5. ^{1}H NMR (400 MHz, 298 K, C_6D_6) spectrum of compound **2a**.

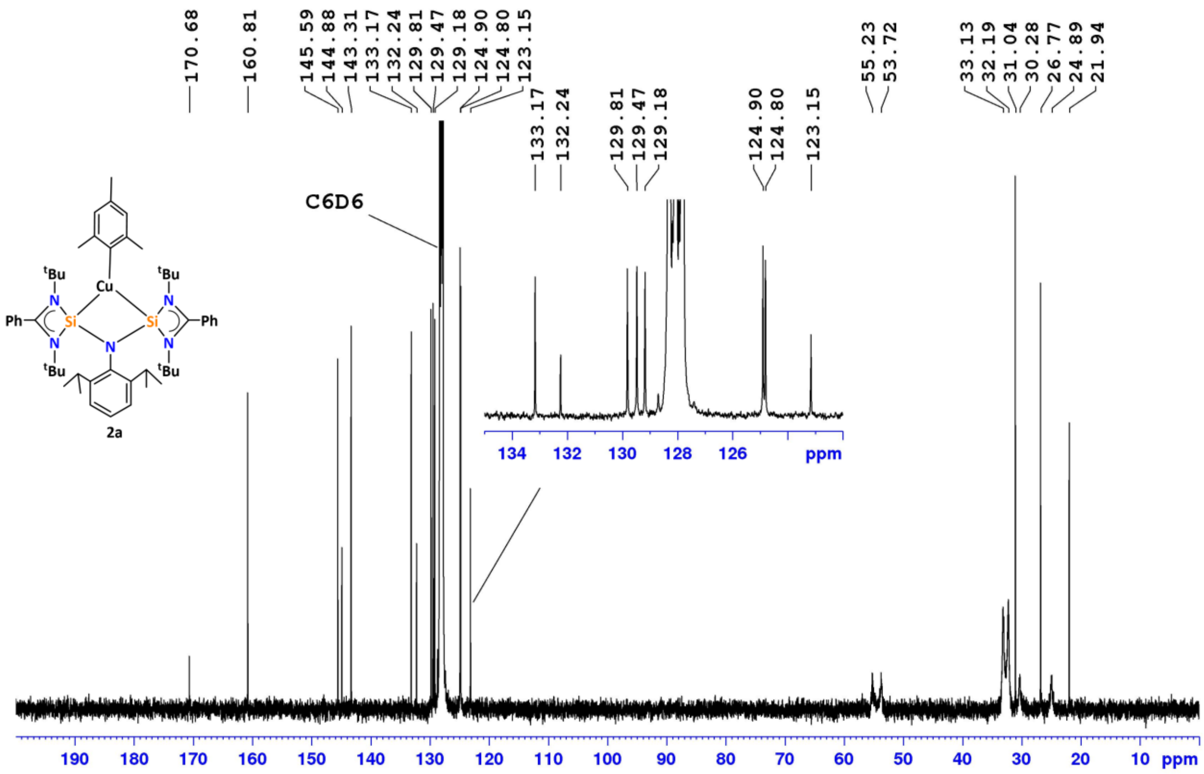

Figure S6. $^{13}C\{^1H\}$ NMR (100 MHz, 298 K, C_6D_6) spectrum of compound **2a**. Signal at 30.3 ppm is due to some unidentified impurity.

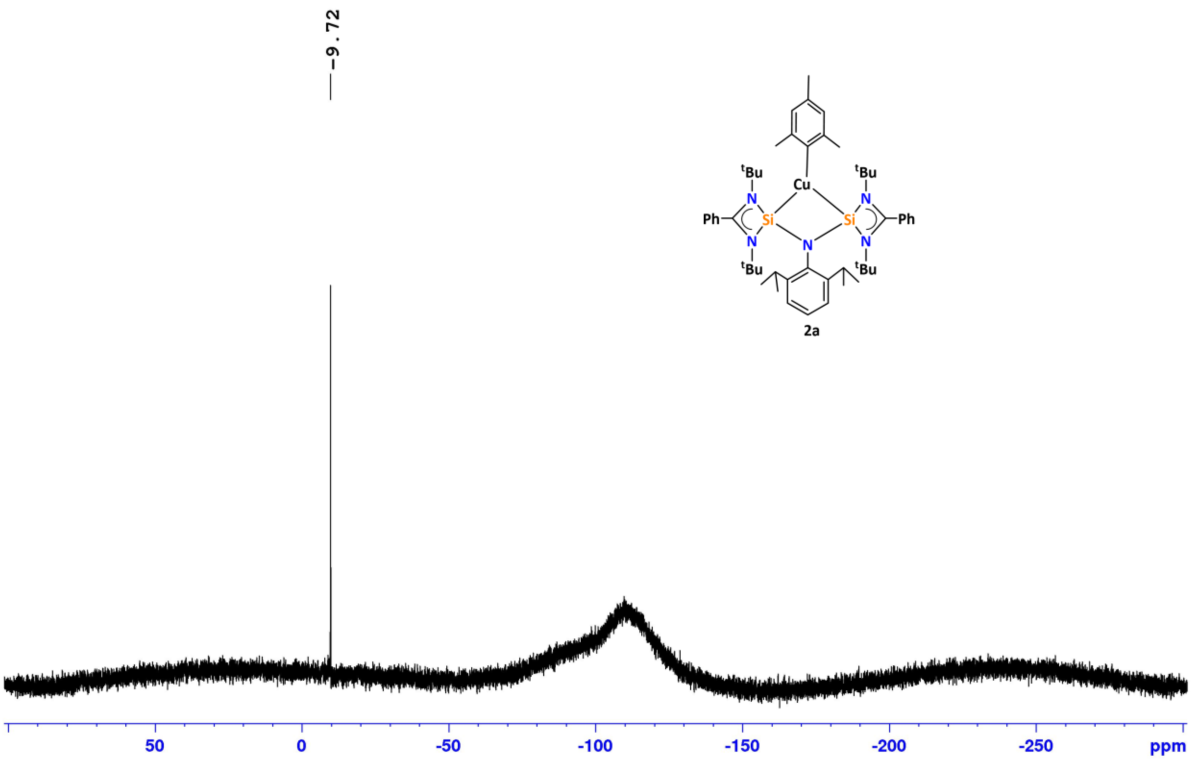

Figure S7. $^{29}Si\{^1H\}$ NMR (79.5 MHz, 298 K, C_6D_6) spectrum of compound **2a**.

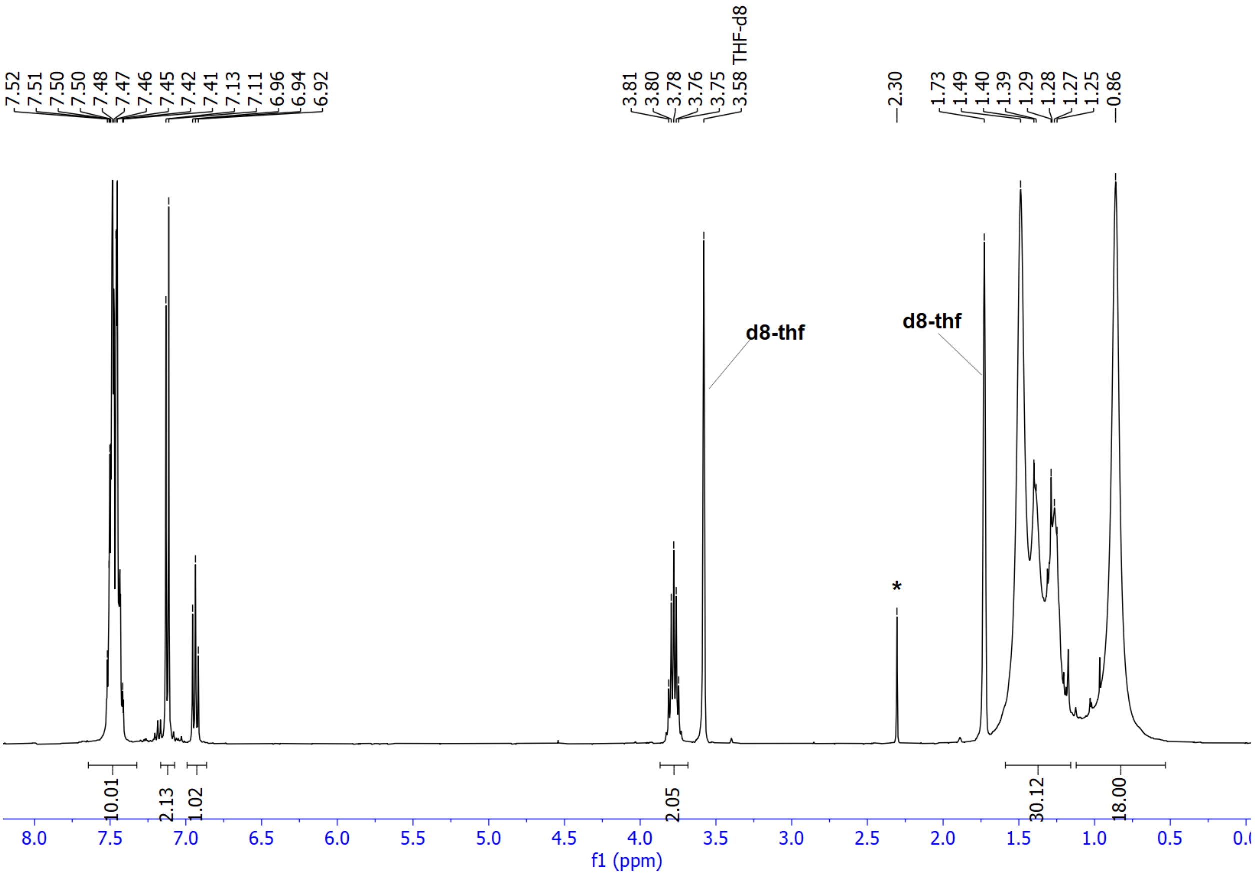

Figure S8. ^{1}H NMR (400 MHz, 298 K, d_8-thf) spectrum of compound **2b**. ***** = residual toluene.

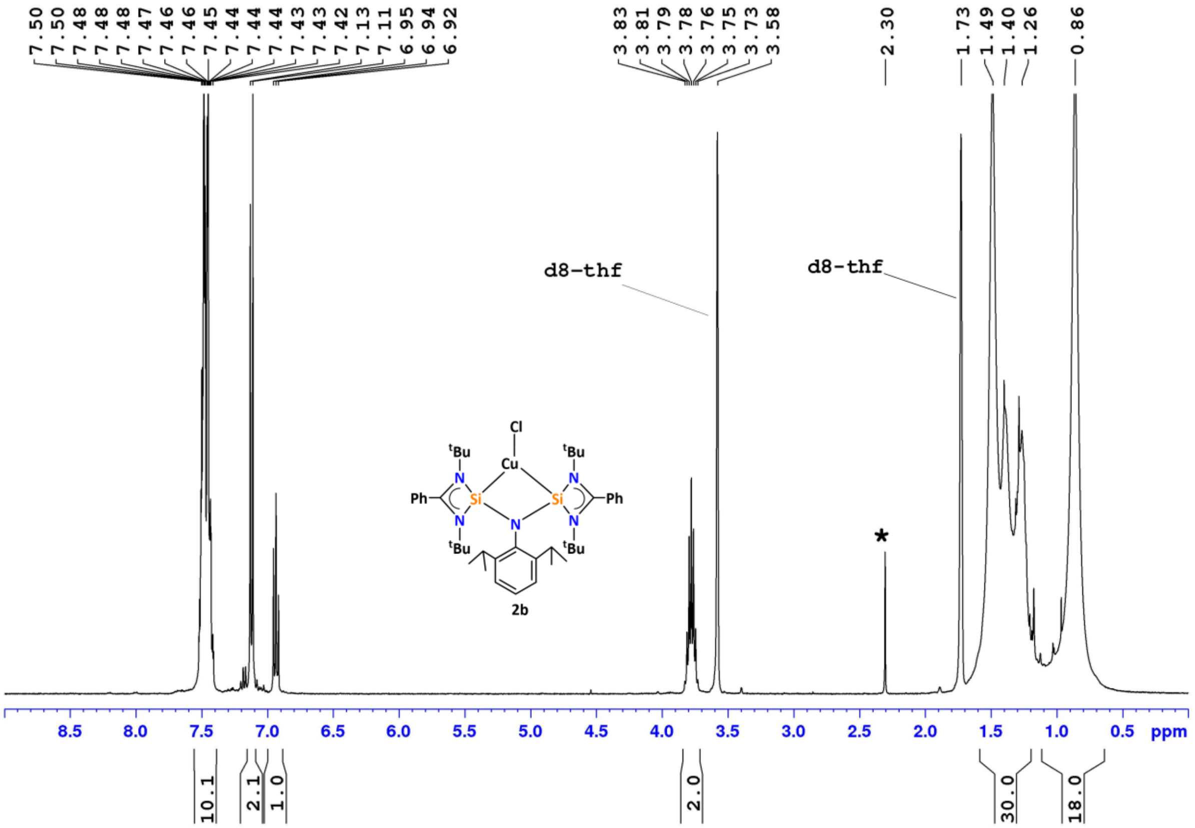

Figure S9. Zoomed-in ^{1}H NMR (400 MHz, 298 K, d_8-thf) spectrum of compound **2b**. * = residual toluene.

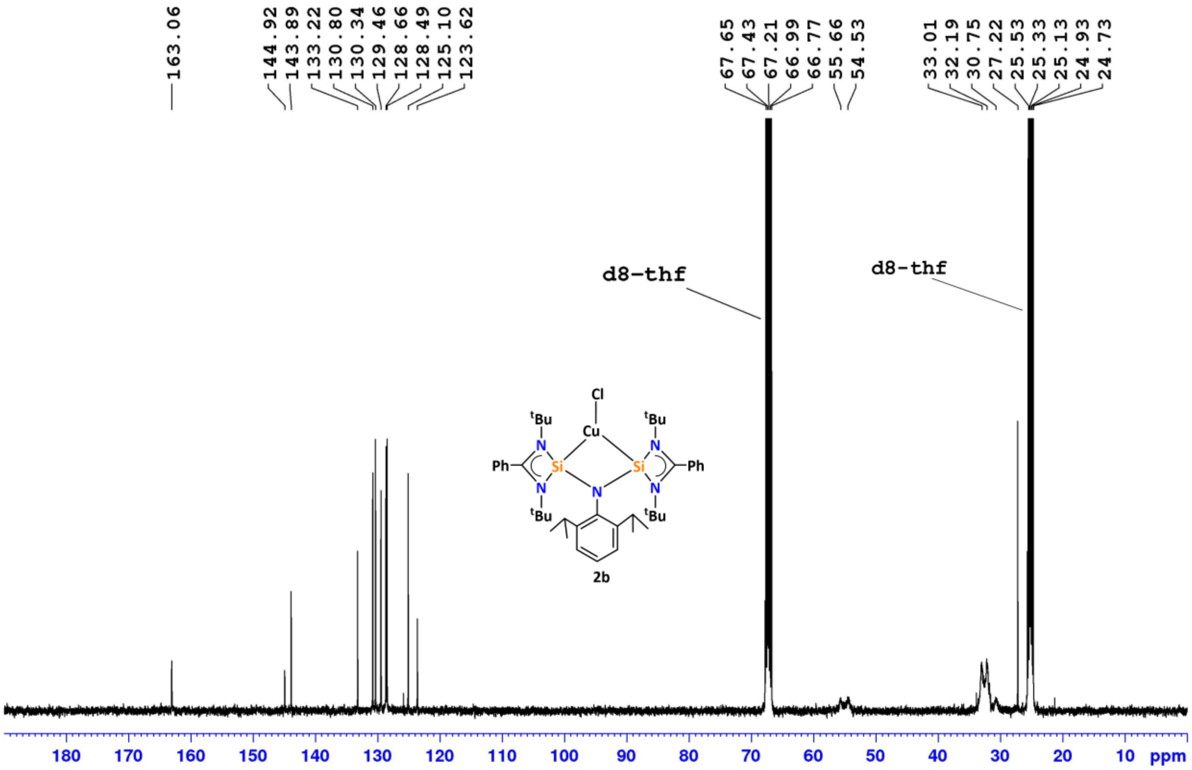

Figure S10. $^{13}C\{^{1}H\}$ NMR (100 MHz, 298 K, d_8-thf) spectrum of compound **2b**.

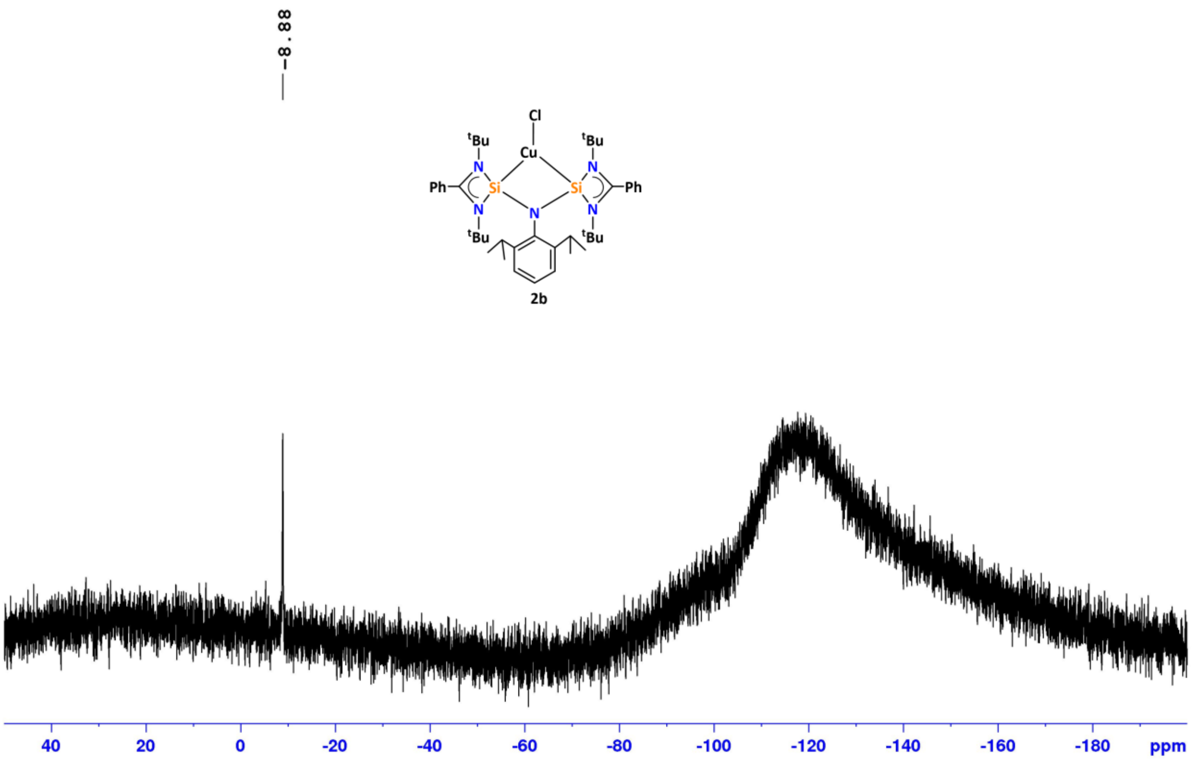

Figure S11. $^{29}Si\{^1H\}$ NMR (79.5 MHz, 298 K, d_8-thf) spectrum of compound **2b**.

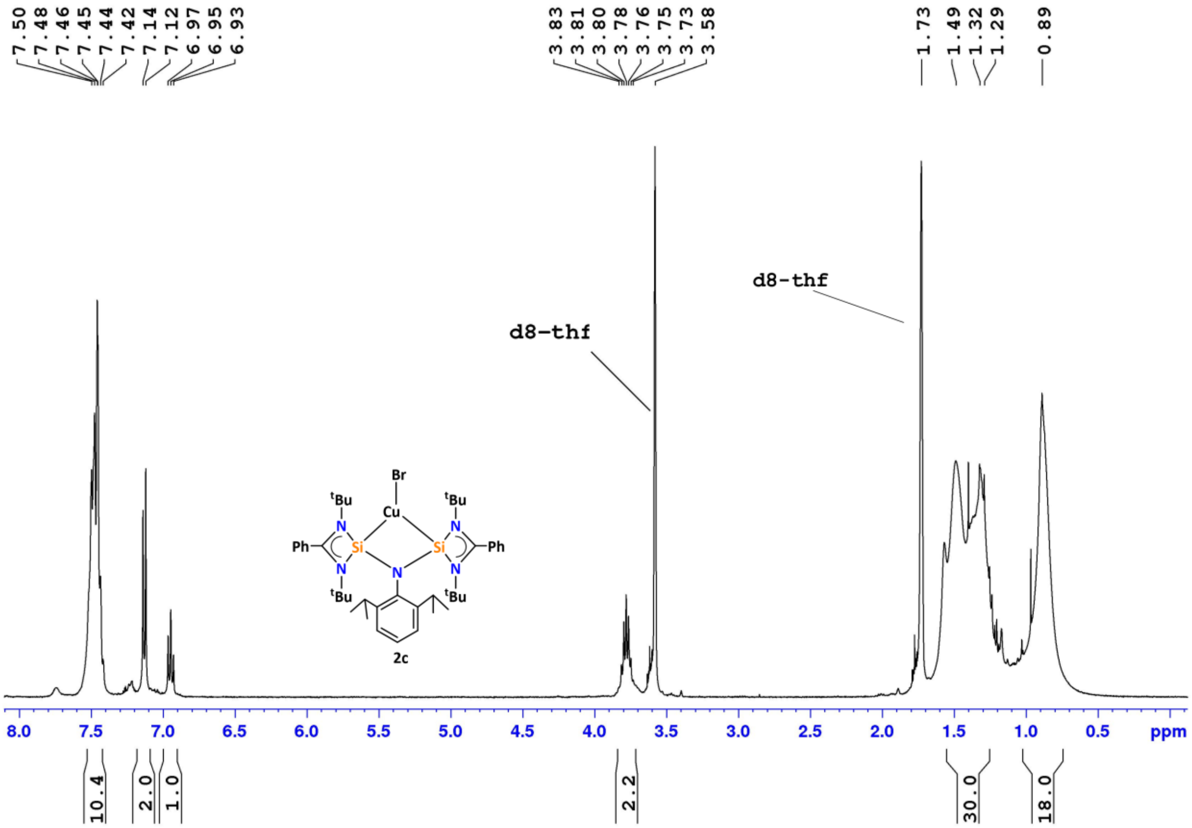

Figure S12. ^{1}H NMR (400 MHz, 298 K, d_8-thf) spectrum of compound **2c**.

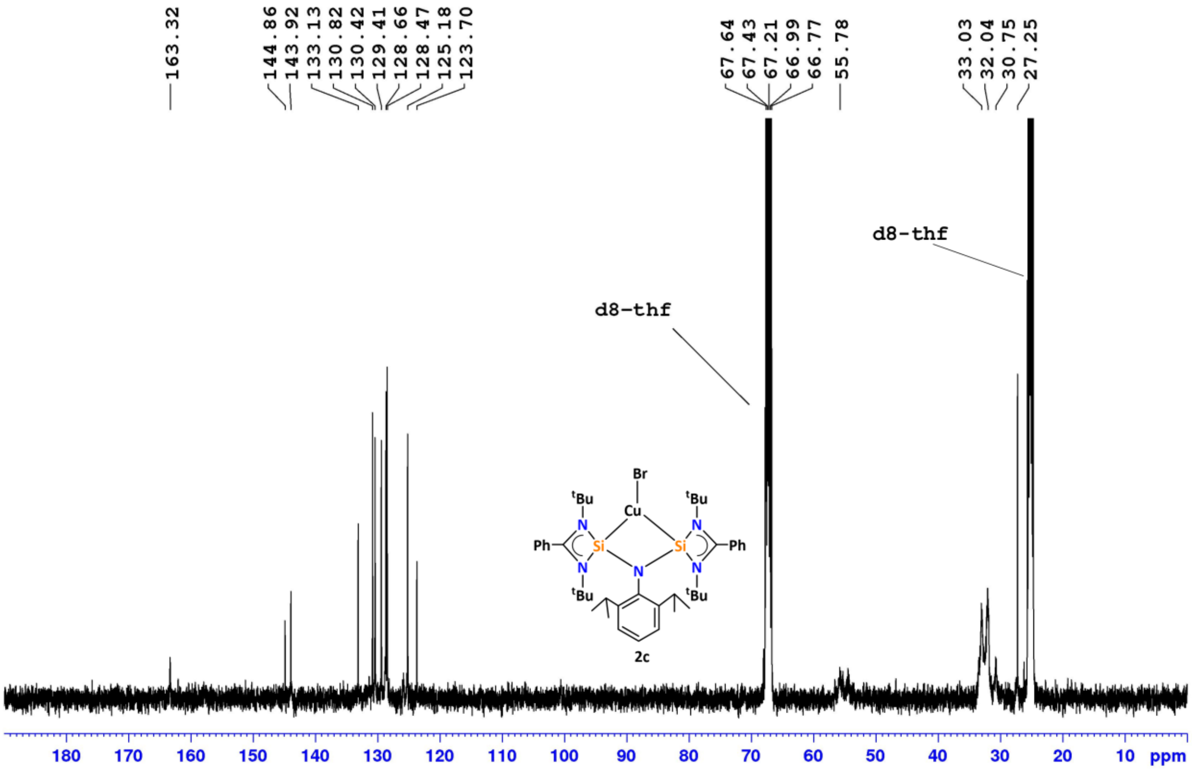

Figure S13. $^{13}C\{^1H\}$ NMR (100 MHz, 298 K, d_8-thf) spectrum of compound **2c**.

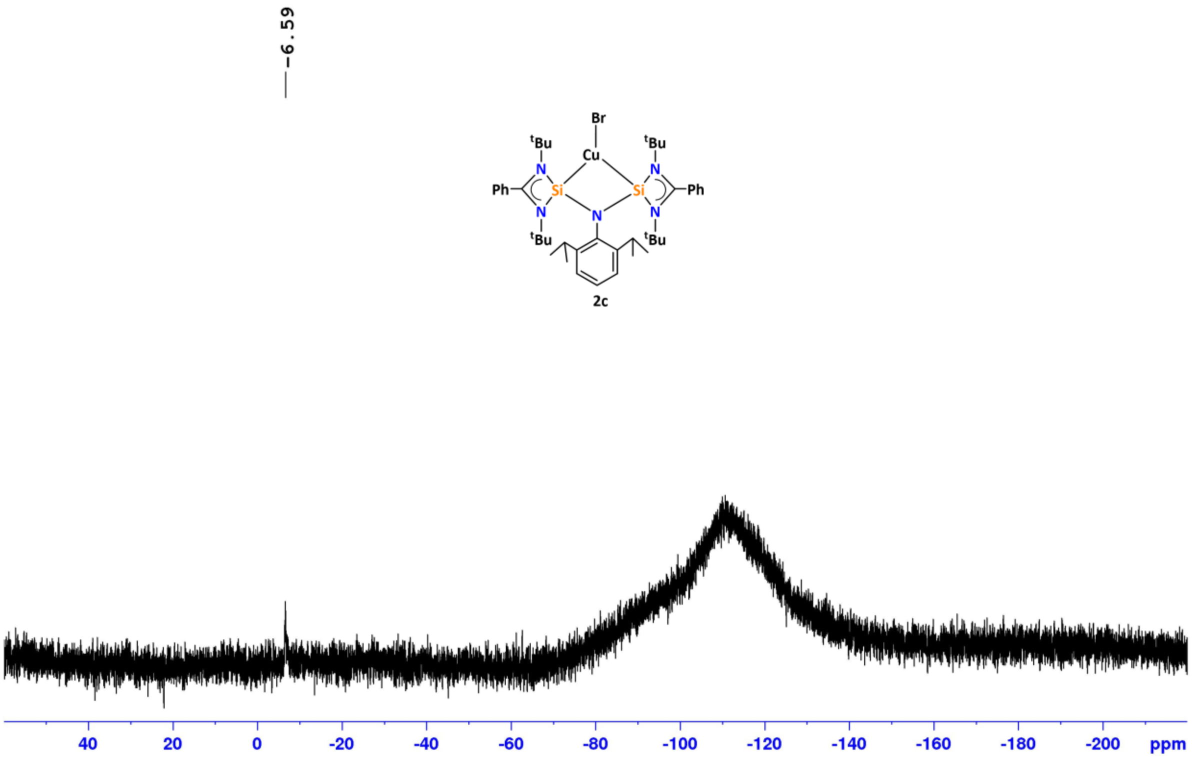

Figure S14. $^{29}Si\{^{1}H\}$ NMR (79.5 MHz, 298 K, d_8-thf) spectrum of compound **2c**.

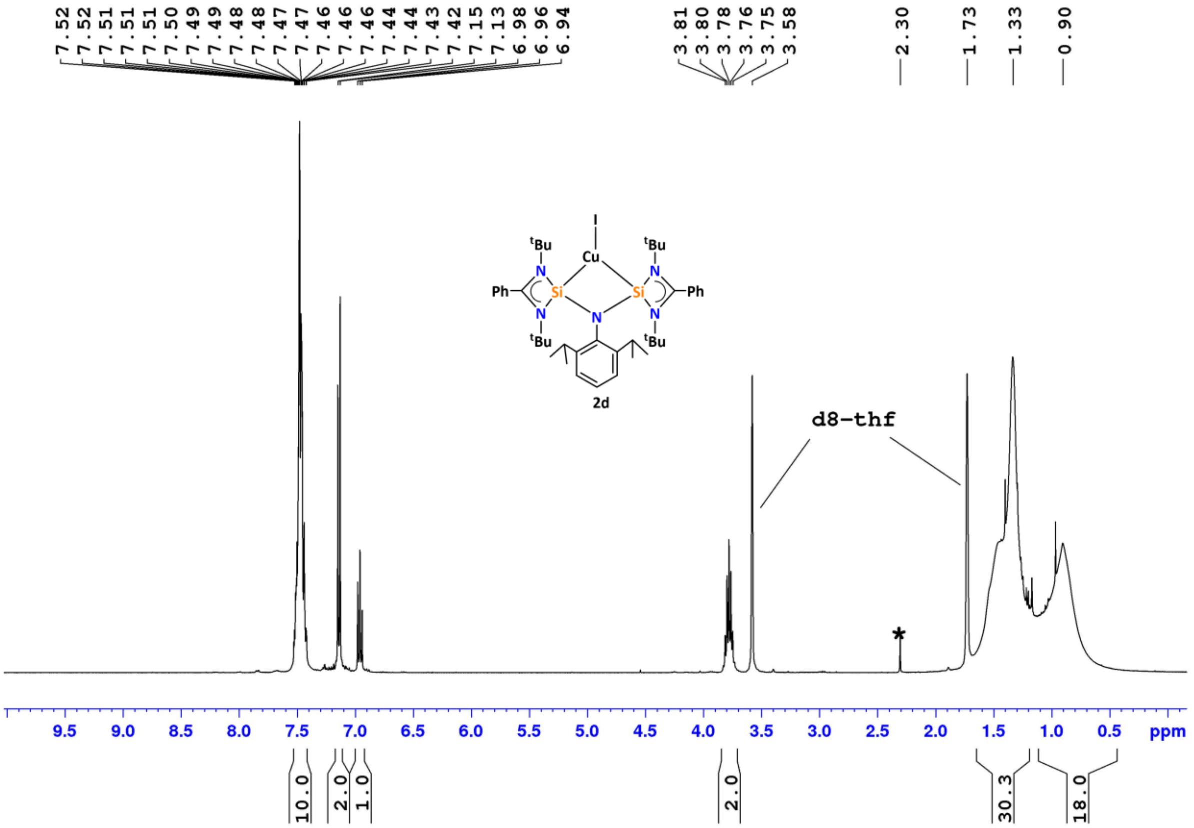

Figure S15. ^{1}H NMR (400 MHz, 298 K, d_8-thf) spectrum of compound **2d**. * = residual toluene.

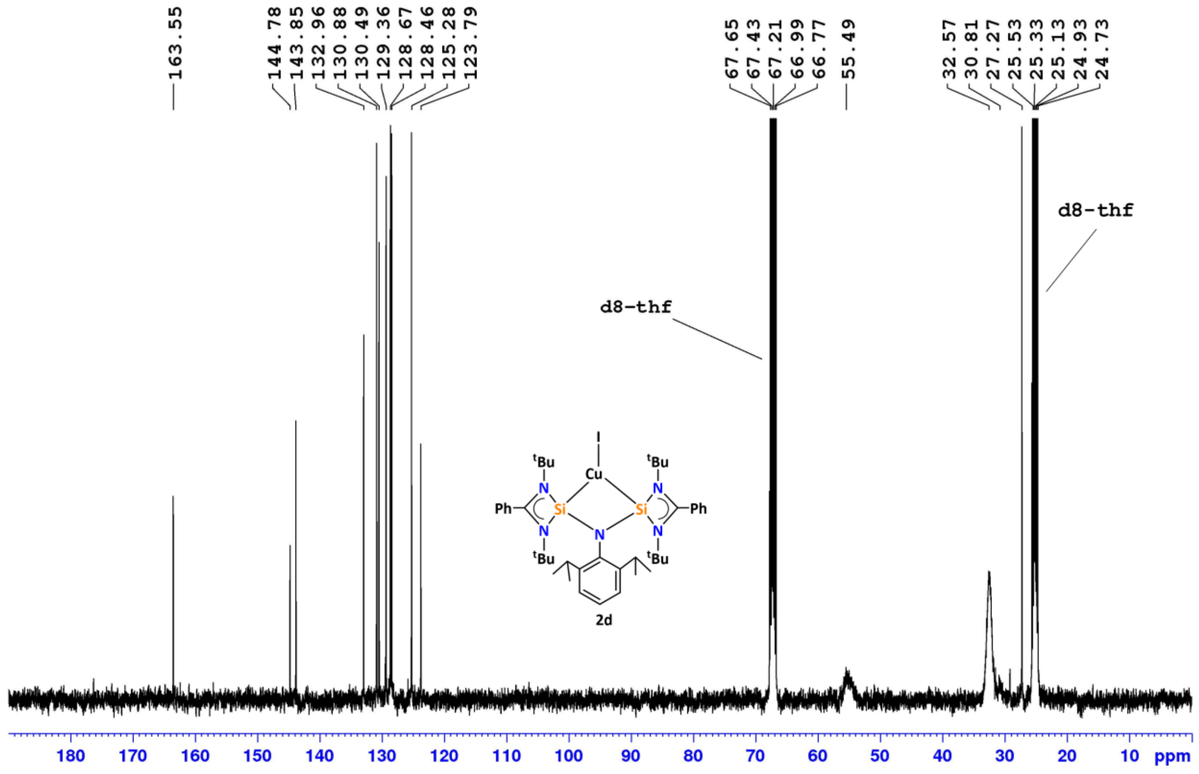

Figure S16. $^{13}C\{^1H\}$ NMR (100 MHz, 298 K, d_8-thf) spectrum of compound **2d**.

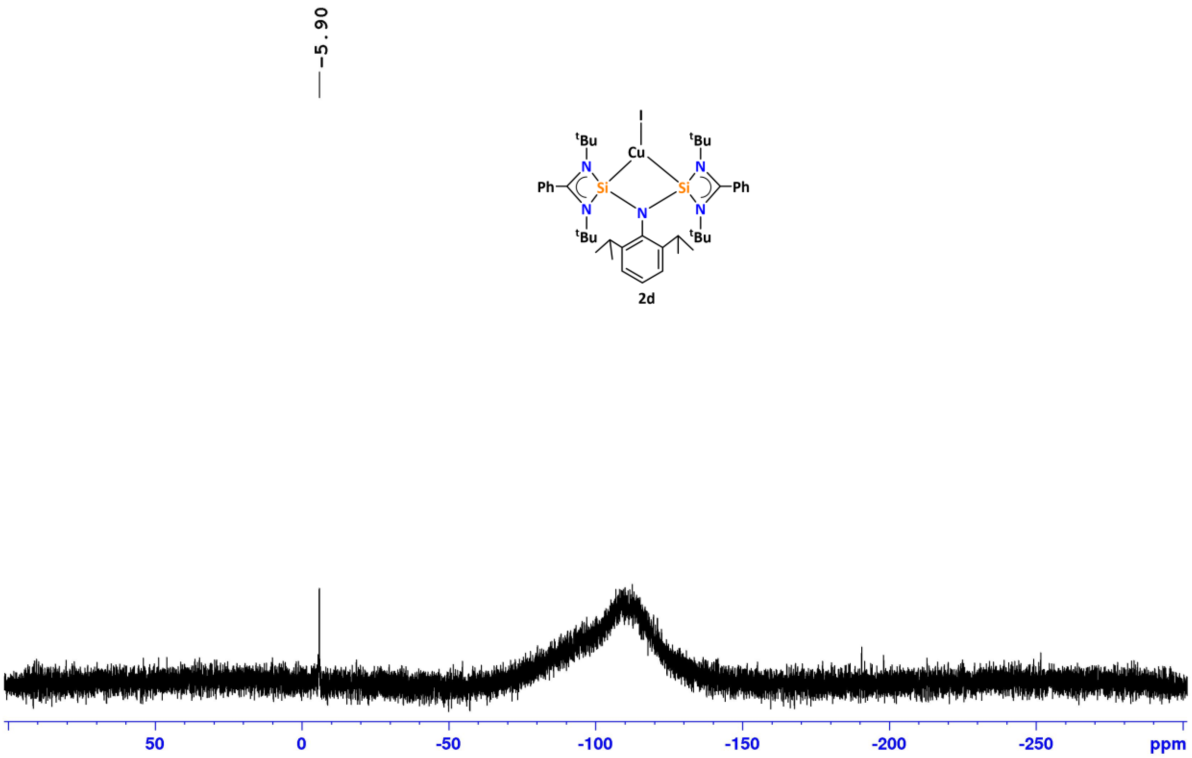

Figure S17. $^{29}Si\{^{1}H\}$ NMR (79.5 MHz, 298 K, d_8-thf) spectrum of compound **2d**.

3. NMR experiments for conversion of 2d to 1 upon adding ITMe (1,3,4,5-tetramethylimidazol-2-ylidene)

In a J-Young NMR tube **2d** (20 mg, 0.022 mmol) and ITMe (5.62 mg, 0.044 mmol) were added and C_6D_6 was added to the mixture. After 1 h the NMR spectra showed the complete conversion of **2d** to **1** along with insoluble precipitates (likely [ITMe-CuI]). The signals corresponding to [ITMe-CuI] could not be observed due to its insolubility. Only one equivalent of ITMe is consumed for the reaction. The NMR below is showing the one equivalent of free ITMe along with **1**. This also shows that compound **1** is stable against ITMe.

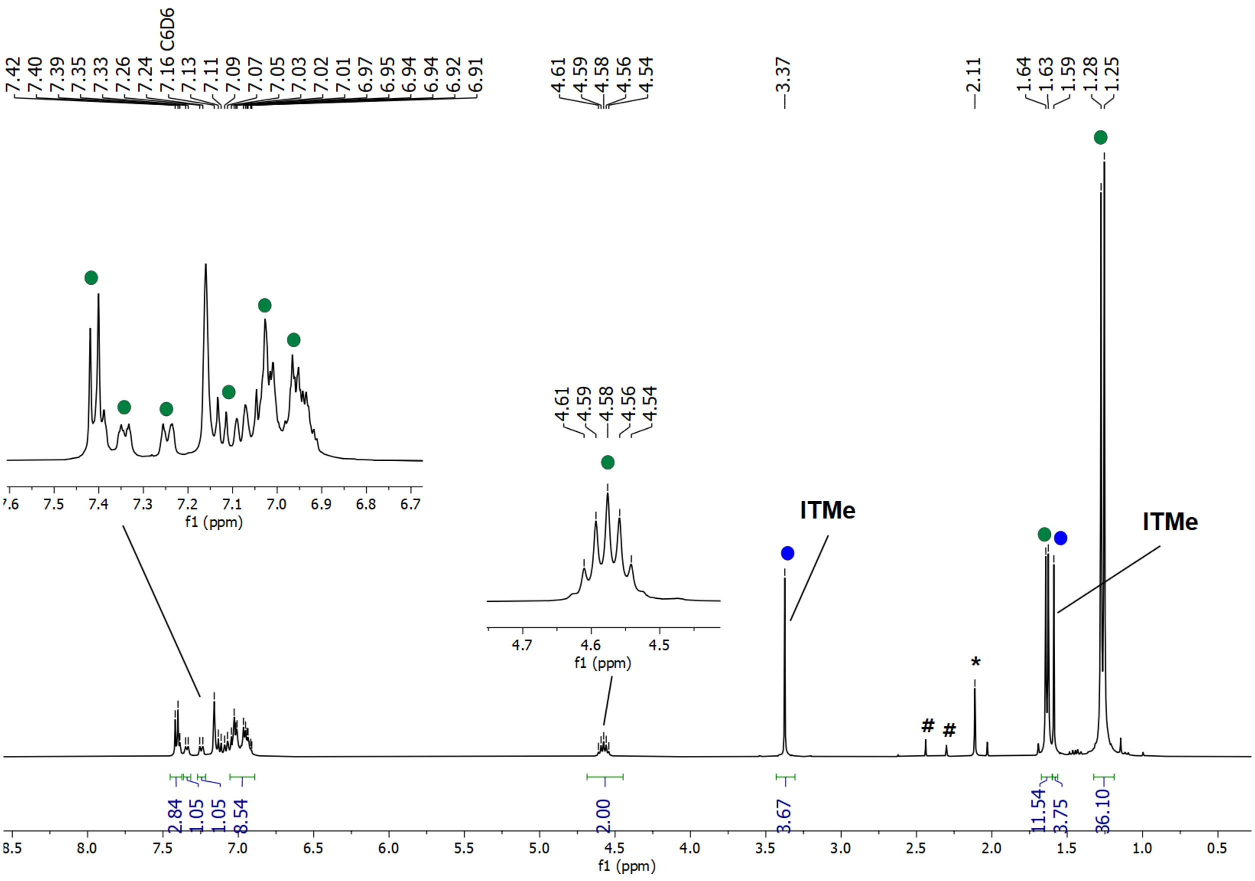

Figure S18. ^1H NMR (400 MHz, 298 K, C_6D_6) spectrum of reaction between **2d** and 2 equivalents of ITMe. The green dots represent **1** and the blue ones represent unconsumed ITMe. * = residual toluene, # = traces of unidentified product(s).

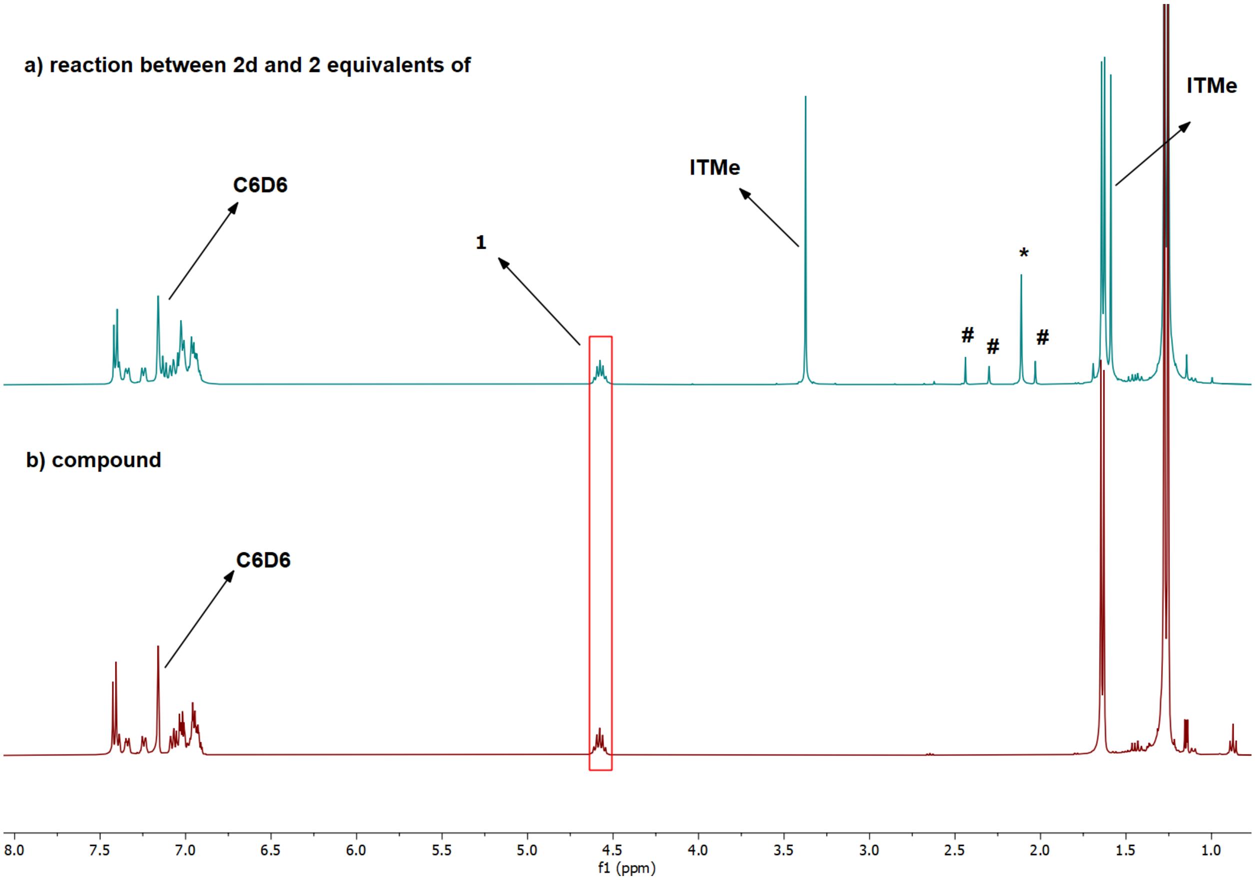

Figure S19. A stack of ^{1}H NMR spectra of a) reaction between **2d** and 2 equivalents of ITMe and b) compound **1**.

4. Single crystal X-ray diffraction analysis

A suitable crystal was covered in mineral oil (Aldrich) and mounted on a glass fiber. The crystal was transferred directly to the cold stream of a STOE IPDS 2 or a STOE StadiVari diffractometer. All structures were solved by using the program SHELXS/T[4] and Olex2.[5] The remaining non-hydrogen atoms were located from successive difference Fourier map calculations. The refinements were carried out by using full-matrix least-squares techniques on F2 by using the program SHELXL.[4] In each case, the locations of the largest peaks in the final difference Fourier map calculations, as well as the magnitude of the residual electron densities, were of no chemical significance.

Crystallographic data for the structures reported in this paper have been deposited with the Cambridge Crystallographic Data Centre as a supplementary publication no. 2192922-2192925. Copies of the data can be obtained free of charge on application to CCDC, 12 Union Road, Cambridge CB21EZ, UK (fax: (+(44)1223-336-033; email: deposit@ccdc.cam.ac.uk).

Comment to compound **2c**:

After completion of the structure refinement, two relatively large residual electron densities remain, which can be assigned to a Cu-Br fragment with identical bond length to the actual target molecule. The chemical occupation is about 4%. This suggests that these are atom positions of an unresolved twin molecule, in which the positions of the lighter remaining atoms cannot be assigned due to too low electron density and overlaps. This not obvious twinning was overlooked during the measurement of the crystal, but also afterwards a twin integration and the search for a suitable twin law fails.

4.1. Table S1 Crystal data and structure refinement

Compound	2a·0.5(Pentane)	2b·3 (Toluene)	2c·2(THF)	2d·2 (Toluene) 0.5(Toluene, Pentane)
Formula	$C_{53.5}H_{80}CuN_5Si_2$	$C_{63}ClCuH_{87}N_5Si_2$	$C_{50}H_{79}BrCuN_5O_2Si_2$	$C_{56}H_{79}CuIN_5Si_2$
$D_{calc.}$/ g cm^{-3}	1.135	1.208	1.293	1.162
μ/mm^{-1}	0.491	0.500	1.315	0.938
Formula Weight	912.94	1069.54	981.81	1068.86
Colour	red	brownish red	red	orange
Shape	fragment-shaped	prism-shaped	fragment-shaped	prism-shaped
Size/mm³	0.40×0.34×0.23	0.27×0.22×0.13	0.43×0.33×0.16	0.25×0.19×0.12
T/K	150	100	100	150
Crystal System	monoclinic	monoclinic	monoclinic	monoclinic
Space Group	*C*2/*c*	*C*2/*c*	*P*2$_1$/*n*	*I*2/*a*
a/Å	22.7217(6)	24.2079(10)	12.1252(17)	21.101(2)
b/Å	15.3128(5)	13.8167(8)	24.928(4)	14.1031(10)
c/Å	32.1035(8)	21.5824(10)	17.358(3)	22.130(2)
α/°	90	90	90	90
β/°	106.914(2)	125.424(3)	105.980(11)	111.975(8)
γ/°	90	90	90	90
V/Å³	10686.7(5)	5882.4(5)	5043.8(13)	6107.2(11)
Z	8	4	4	4
Z'	1	0.5	1	0.5
Wavelength/Å	0.71073	0.71073	0.71073	0.71073
Radiation type	Mo K$_\alpha$	Mo K$_\alpha$	Mo K$_\alpha$	Mo K$_\alpha$
Θ_{min}/°	1.326	2.415	2.009	1.752
Θ_{max}/°	25.206	31.479	29.480	29.268
Measured Refl.	30150	15473	24875	43658
Independent Refl.	9514	7536	12256	8231
Reflections Used	7875	5709	10103	7014
R_{int}	0.0202	0.0255	0.0207	0.0192
Parameters	551	305	574	305
Restraints	0	0	0	99
Largest Peak	0.674	0.467	1.595	1.572
Deepest Hole	-0.274	-0.225	-0.540	-0.590
GooF	1.039	1.047	1.022	1.041
wR_2 (all data)	0.1140	0.0990	0.1069	0.0916
wR_2	0.1108	0.0938	0.0993	0.0871
R_1 (all data)	0.0505	0.0548	0.0559	0.0397
R_1	0.0417	0.0378	0.0425	0.0324

4.2. Crystal Structures

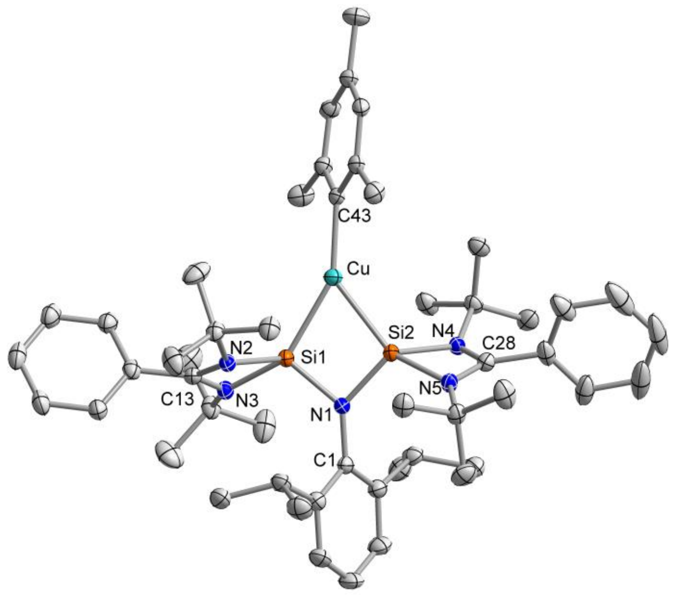

Figure S20. Molecular structure of complex **2a** in the solid state with thermal ellipsoids at 40% probability. The hydrogen atoms and non-coordinated solvents are omitted for clarity. Selected bond lengths (Å) and bond angles [°]: Cu-Si1 2.2801(6), Cu-Si2 2.3355(6), Cu-C43 1.961(2), Si1-N1 1.7850(2), Si1-N2 1.8880(2), Si2-N1 1.7930(2), Si1-N3 1.840(2), Si2-N4 1.8950(2), Si2-N5 1.8420(2), Si···Si 2.6149(8); Si1-Cu-Si2 69.01(2), N1-Si2-Cu 97.24(6), Si1-N1-Si2 93.87(8), N2-C13-N3 105.60(2), N5-C28-N4 106.15(2).

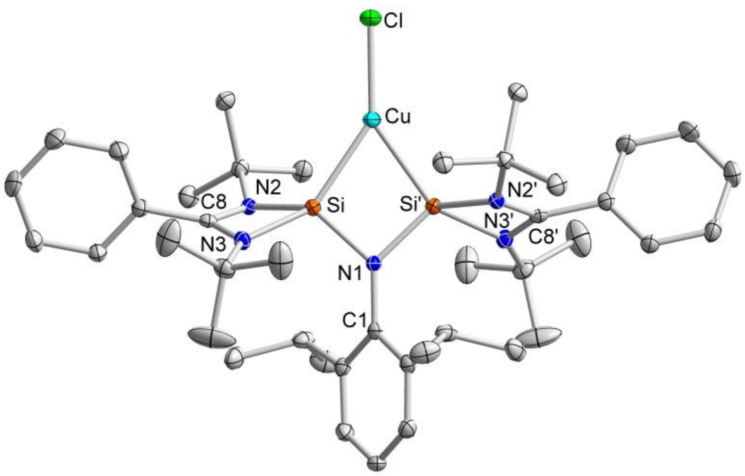

Figure S21. Molecular structure of complex **2b** in the solid state with thermal ellipsoids at 40% probability. The hydrogen atoms and non-coordinated solvents are omitted for clarity. Selected bond lengths (Å) and bond angles [°]: Cu-Cl 2.1797(6), Cu-Si' 2.2847(5), Cu-Si 2.2846(5), Si-N1 1.7837(13), Si-N2 1.8750(13), Si-N3 1.8193(13), N1-C1 1.449(3), N2-C8 1.3460(2), N3-C8 1.3420(2), N3-C19 1.4760(2), Si···Si' 2.6187(8); Si-Cu-Si' 69.94(2), Si-N1-Si' 94.45(9), N3-C8-N2 105.14(12).

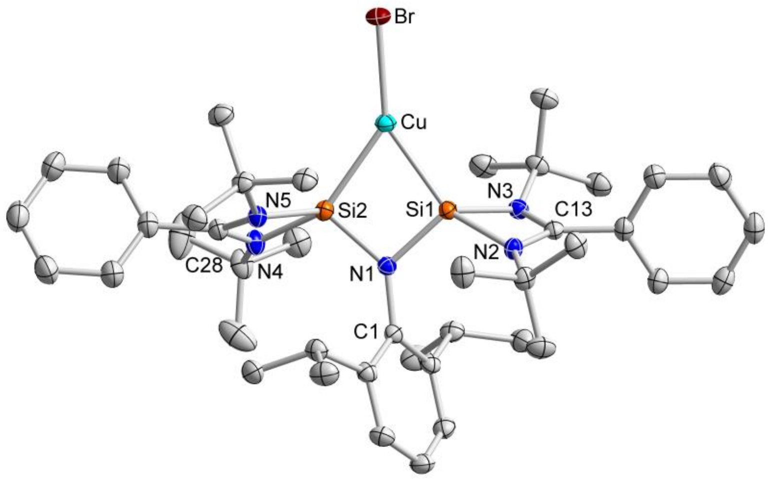

Figure S22. Molecular structure of complex **2c** in the solid state with thermal ellipsoids at 40% probability. The hydrogen atoms and non-coordinated solvents are omitted for clarity. Selected bond lengths (Å) and bond angles [°]: Cu-Br 2.3108(5), Cu-Si1 2.2762(7), Cu-Si2 2.3099(7), Si1-N1 1.7870(2), Si1-N2 1.8230(2), Si1-N3 1.884(2), Si2-N1 1.7840(2), Si2-N4 1.835(2), Si2-N5 1.881(2), N1-C1 1.453(3), N2-C13 1.343(3), N4-C28 1.347(3), N5-C28 1.346(3); Si1-Cu-Si2 71.10(2), Si2-N1-Si1 96.64(9), N3-C13-N2 105.90(2), N5-C28-N4 105.60(2).

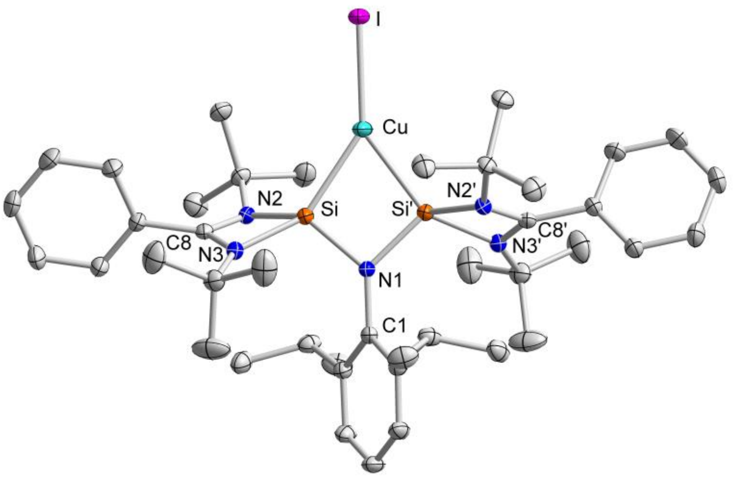

Figure S38. Molecular structure of complex **2d** in the solid state with thermal ellipsoids at 40% probability. The hydrogen atoms and non-coordinated solvents are omitted for clarity. Selected bond lengths (Å) and bond angles [°]: Cu-I 2.4746(4), Cu-Si 2.2887(5), Cu-Si’ 2.2887(5), Si···Si’ 2.6432(9), Si-N1 1.7863(12), Si-N2 1.8828(14), Si-N3 1.8228(14), N1-C1 1.451(3), N3-C8 1.345(2); Si-Cu-I 144.729(11), Si-Cu-Si 70.54(2), N1-Si-N2 119.74(5), Si-N1-Si’ 95.44(9), N2-C8-N3 105.60(13).

5. Quantum chemical calculations

To investigate the bonding properties in the system under discussion [**L**Si-Si(NDipp)**L**] **1**, [**L**Si-Si(NPh)**L**], **2d**, CuI, ITMe and ITMe-CuI, quantum chemical RI-DFT calculations were performed using the BP-86 functional.[6] The basis sets were of def-SVP quality for all atoms as given in the program package TURBOMOLE.[7] For iodine an effective core potential (ecp) of 46 core electrons was chosen. Calculations were performed under the constraint of symmetry C_2 for the (bis) silylene compounds and complex **2d**, otherwise without symmetry constraints. The reaction progress between **1** and **II** were calculated in a preliminary way using the same calculation method (Figure S39).

The Si NMR shifts of **1**, **II** and **2d** were calculated using the mpshift module[8] of the TURBOMOLE program package. Tetramethylsilane (TMS) was taken as a reference substance in the calculation. Partial charges were determined using the Ahlrichs-Heinzmann population analysis based on occupation numbers.[9] Contour plots of the electronic charge density and bond critical points were obtained using the program multiwfn.[10]

The transformation pathway for the isomerization of **II** to **1** was pre-optimized with the corresponding tool in TURBOMOLE, a chain-of-states method that optimizes reaction paths under the sole constraint of equally spaced structures[11] by employing 79 intermediate structures. The resulting pathway is shown in Fig. S39 and also available as movie in file path.mp4. Starting from **II**, at first NDipp moves via **TS1** from the middle of the Si-Si bond to one of the Si atoms (**LM1**), then the L unit at the other Si rotates to give **1**. Final optimizations of stationary points were done with a fine grid (grid 5)[12] and employing weight derivatives, as well as with auxiliary basis sets of higher quality[13] and a tight self-consistent field (SCF) threshold of $10^{-9}E_h$ for the energy and 10^{-4} for the gradient norm in the structure optimizations. This yields the relative energies shown in Table S2. For **II**/**LM1**/**1** the lowest frequencies amount to 20/16/14 cm^{-1}, for **TS1**, one imaginary frequency was found amounting to 102i cm^{-1}. The second part of the transformation exhibits rather a plateau than a sharp transition state due to the coupled movement of the Si-N-C-N ring and the ligands, see Fig. S39. Even by thorough optimization it was not possible to achieve a gradient norm below $4*10^{-4}$ and also not possible to separate the corresponding two modes with low imaginary frequencies. The mode dominated by the rotation of the Si-N-C-N ring amounts to 14i cm^{-1}, the one rather dominated by rotations within the ligands to 8i cm^{-1}. The energy for this state, **TS2**, is lower than that for **TS1**. Thus, surmounting the latter is the determining step. For the optimized structures thermal contributions for T=298.15K were calculated within the harmonic oscillator rigid rotor model[14] with frequencies scaled by 0.9914. Further, for these structures single point calculations with the same strict settings were done with B3LYP[15]/def2-TZVP[16] without and with COSMO[17] for modelling the solvent (epsilon=2.4 for toluene), and with the D3[18] correction for dispersive interactions.

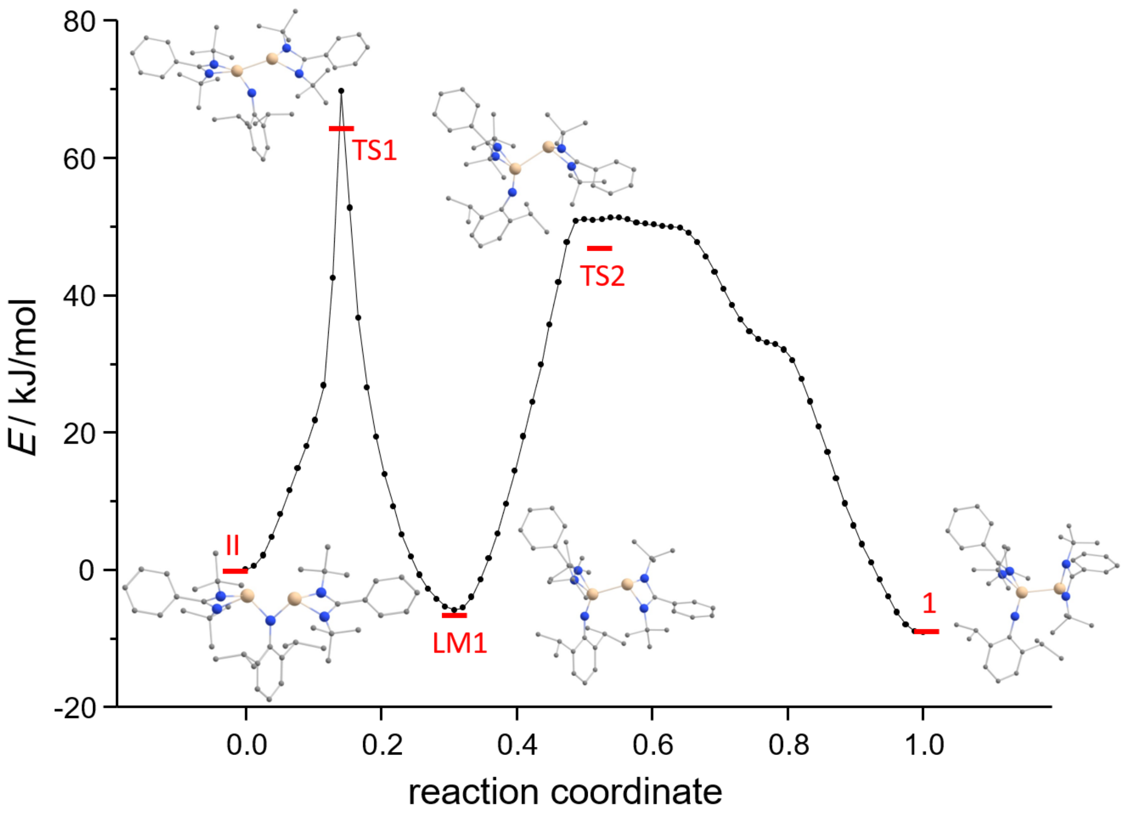

Figure S39. Reaction pathway for the isomerization of **II** to **1**. Hydrogen atoms are omitted for clarity. The red bars indicate the energetic positions after individual optimization of the stationary points. For details see text.

Table S2. Energies for **TS1**, **LM1**, **TS2** and **1** relative to **II**. ΔH denotes the enthalpy contribution at 298.15 K (frequencies scaled by 0.9914, see also Table S5). For details see text.

	BP-86 def-SVP	BP-86 def-SVP +ΔH	BP-86 def-SVP COSMO	BP-86 def2-TZVP	BP-86 def2-TZVP +D3	B3-LYP def2-TZVP	B3-LYP def2-TZVP COSMO(ε=2.4)
**TS1**	+63.3	+59.8	+62.8	+60.2	+75.9	+66.7	65.6
**LM1**	-5.9	-5.2	-8.9	-7.7	+5.0	-16.5	-19.8
**TS2**	+36.9	+34.5	+32.2	+35.6	+59.2	+25.4	+18.0
**1**	-9.3	-9.4	-15.2	-11.3	+4.3	-22.2	-28.9

Table S3. Important experimental and theoretical distances (given in Å) of the molecule **2d**.

**2d**	Exp.	Theory
r(Cu-I)	2.475	2.518
r(Cu-Si)	2.289	2.303
r(Si…Si)	2.643	2.669
r(Si-N1)	1.786	1.835
r(Si-N2)	1.893	1.931
r(Si-N3)	1.823	1.884
r(N1-C1)	1.451	1.440

Table S4. Important experimental and theoretical distances (given in Å) of the molecule **1**.

**1**	Exp.	Theory
r(Si(I)-Si(III))	2.364	2.421
r(Si(I)-N)	1.870/1.866	1.915/1.923
r(Si(III)-N)	1.852/1.865	1.952/1.909
r(Si(III)=N)	1.592	1.625

Table S5. Total energies, lowest vibration frequencies and enthalpy at 298.15 K (frequencies scaled by 0.9914) of the molecules under discussion.

Compound	Total energy/a.u.	Lowest frequencies/(cm^{-1})	H/(kJ mol^{-1})
**1**	-2489.784524	14/15/20	2607.2
**TS1**	-2489.756878	102i/17/19	2603.7
**LM1**	-2489.783235	16/17/23	2607.9
**TS2**	-2489.766093	14i/8i/7	2604.8
**II**	-2489.780998	20/23/25	2627.1
**2d**	-4141.940398	15/22/23	2625.1
CuI	-1652.044025	256	11.5
ITMe	-383.175679	124/126/146	489.2
ITMe-CuI	-2035.333309	31/35/111	507.5

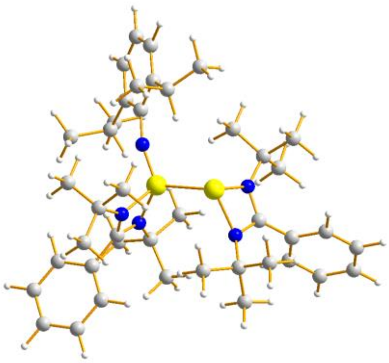
a)

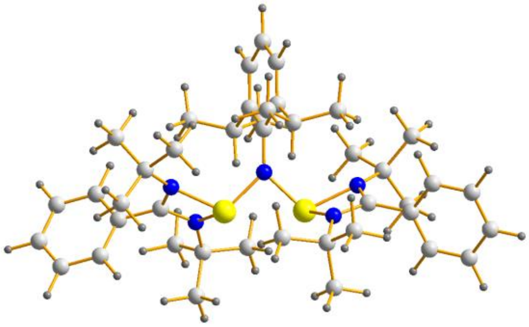
b)

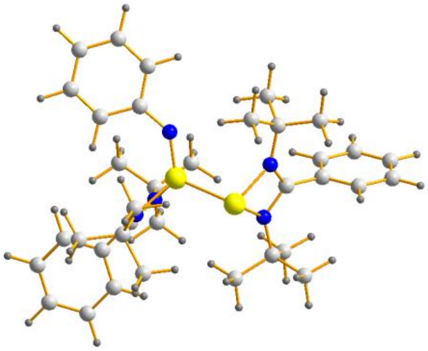
c)

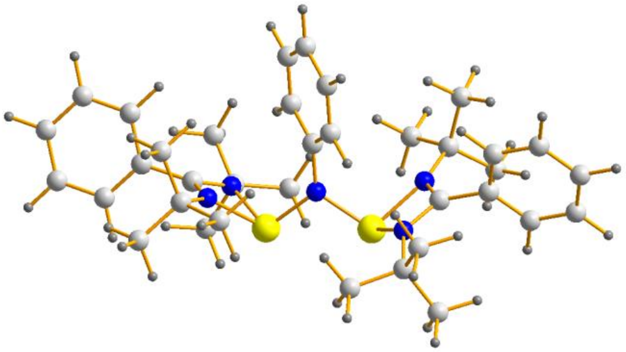
d)

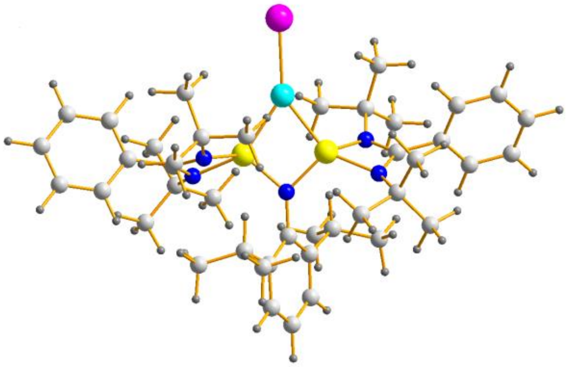
e)

Figure S40. Calculated molecular structures of a) **1**, b) **II** [**L**Si-N(Dipp)-Si**L**], c) [**L**Si-Si(NPh)**L**], d) isomer **II** [**L**Si-N(Ph)-Si**L**] of [**L**Si-Si(NPh)**L**] and e) **2d** (nitrogen, silicon, carbon and hydrogen atoms are given in blue, yellow, large grey and small grey).

Cartesian Coordinates of the molecules under discussion (given in a. u.):

[**L**Si-Si(NDipp)**L**] **1:**

-2.80884859839279	-2.51312150182660	2.10597614199788	n
1.90458364230254	-2.68742167994968	-1.31858462685731	n
-1.81090659677123	4.67063150579444	-1.26334657419506	n
2.70265717977740	-2.52356148672876	2.72529067054231	n
2.17934732535018	4.96633122642410	-0.40037320795284	n
-0.24065011237798	-1.08278907073578	1.21568010742221	si
-0.29438944594039	3.45504073288816	1.78991449540129	si
3.57824135208855	-3.35374773373151	0.47966663465863	c
0.35161013625267	5.81465593436181	-1.99826136456650	c
2.14952889649224	-2.60350057900785	-4.10601088931262	c
-0.29048537792145	-1.35903294380959	-5.10064481105713	c
-1.97398977379445	-2.49707478680564	-4.59698152790769	h
-0.21788254365895	-1.18927883347557	-7.18682501262007	h
-0.53161720757306	0.55569249754593	-4.28319672575816	h
4.43150916022815	-0.97677788379929	-4.92121538909989	c
2.36807199260282	-5.29118498397224	-5.22452006544636	c
4.29112578696884	0.93325566499694	-4.07205710518914	h
4.15926707157832	-6.22085104536814	-4.67619456730373	h
4.46920316220027	-0.77190862697094	-7.00744008204862	h
2.31354315028711	-5.19875525858455	-7.31779948346814	h
6.24801324733912	-1.83620184403101	-4.33875711012492	h
0.77365484691181	-6.48736192339010	-4.58340022527164	h
-4.46485402472061	4.94739513215614	-2.11861677965624	c
3.29006772816257	-3.28453911777505	5.35865273197942	c
4.70259299958122	6.02981139699417	0.18778032129079	c
-5.90048497245220	2.60452517992927	-1.15781964967343	c
2.17249292059596	-1.21129569040817	7.07601383715712	c
5.89674367295021	4.22350104094270	2.13677208952124	c
-5.18348708938650	0.84665442163316	-2.03552681434588	h
0.11668255458780	-0.99921855671608	6.75094356688360	h
6.07913291794036	2.29101827234970	1.35144619131698	h
-7.94114946115624	2.76915398389803	-1.59412131658774	h
2.46672456901896	-1.70576549486788	9.08945375019424	h
7.79507418577915	4.89957276586235	2.70567206568697	h
-5.71027187809359	2.39153202684413	0.91801780744985	h
3.08713732204489	0.63833566229593	6.71386490609172	h
4.70628159273399	4.10847461067687	3.86057550454843	h
-5.63527363052610	7.34352261820617	-0.91936500991562	c
6.15111942388734	-3.48241659849977	5.87951617248869	c

4.44773698918917	8.68423872940111	1.38641687502455	c
-4.68256942276491	5.07487677373783	-5.02339116365418	c
1.98029065127304	-5.83548335077630	5.91985274598791	c
6.42233193375346	6.16009758216766	-2.16409152545247	c
-5.45230211614978	7.28182024128698	1.16631827935957	h
7.14393555575416	-1.71668595403733	5.34545246200393	h
3.18071082319492	8.61170747002045	3.05374136886451	h
-3.85408938993703	6.82325899352247	-5.81468385786048	h
2.78458910101451	-7.36490672795907	4.73409815799313	h
5.71601863307307	7.52406806209164	-3.58168446506039	h
-7.67183501347061	7.47793679197702	-1.39645792299852	h
6.44742857617052	-3.78873675828025	7.93123917911484	h
6.31894545725173	9.39556549386791	2.01006952401460	h
-6.70662252362166	5.03144162554675	-5.56233293609414	h
2.24285039023773	-6.37342727338229	7.92892640693455	h
8.34514537246770	6.77241923063486	-1.59792410187581	h
-4.69954083545518	9.08109094311020	-1.61801313536675	h
7.04721667606159	-5.07784809121472	4.87053086999864	h
3.66814941975201	10.06304180613238	0.01801548648593	h
-3.74729546801353	3.43122074065771	-5.92265248055663	h
-0.06952414826744	-5.69564119598139	5.51851038989242	h
6.58601127600933	4.28363385260572	-3.07960797386692	h
5.98242414254961	-4.78799608481978	0.07009128672811	c
5.90754664551439	-7.44729526240049	-0.14222297186905	c
8.15032968384908	-8.82607800828834	-0.47582910127830	c
10.48388781558580	-7.56755038217803	-0.61934694317374	c
10.56859186396842	-4.92021740073317	-0.42046667686373	c
8.33389298899564	-3.53589504106053	-0.06927697333804	c
4.07975392400763	-8.43264916296288	-0.03606235796927	h
8.07018008572591	-10.89898507652862	-0.62662455350759	h
12.23954944066195	-8.65064356795222	-0.88508574894913	h
12.39064223078526	-3.92298793837100	-0.53042220907237	h
8.40849640225019	-1.46647380395883	0.11247851469354	h
0.65234986360942	7.73431823446421	-4.05284331476320	c
1.57447022668932	6.99291464421005	-6.44691553723209	c
1.84149583019539	8.76904813892317	-8.39830676871783	c
1.19979310346501	11.31317746679198	-7.98487407879205	c
0.29113394076706	12.07251766586956	-5.60856735010338	c
0.01656838892876	10.29701221017802	-3.65465019306148	c
2.06693445139396	4.99966142079668	-6.77374522906377	h
2.55079580778972	8.16082427208463	-10.25688169725585	h
1.40784284568220	12.70511330281428	-9.51650649370635	h
-0.20908813251657	14.06306663708467	-5.26962432420905	h
-0.69110520528174	10.90326757501318	-1.79608284650375	h

-5.27290823182675	-3.22718861916025	2.30774996292792	c
-6.32398487845054	-5.13774880937460	0.65741498915209	c
-8.86336350288246	-5.86563820639302	0.91538555910772	c
-10.44219439636548	-4.79542609614150	2.75878651213120	c
-9.44137920834249	-2.95649745186297	4.38282869397149	c
-6.91773138842864	-2.14368726926065	4.20344854381805	c
-9.63665043970329	-7.32294576669560	-0.35454386761102	h
-12.42759446156621	-5.39358129847128	2.92945410627627	h
-10.66928858917946	-2.12032228412356	5.84276885664947	h
-4.60982659640336	-6.42505225820437	-1.26826613405318	c
-3.18134493589501	-8.65375663011051	-0.04008532699201	c
-4.52147634467501	-10.14342013237182	0.58375498489510	h
-1.82648185592757	-9.53495808569710	-1.38423706812020	h
-2.11177220451328	-8.00645681085725	1.63920661360235	h
-5.95484915267288	-7.26918414964475	-3.70136873395969	c
-3.15622987098756	-5.00225349097757	-1.78861277438936	h
-4.56647713067822	-7.97085237932553	-5.11056512367441	h
-7.02966084029768	-5.69729923925571	-4.57833594295438	h
-7.30553027727537	-8.83775934313284	-3.35530456358195	h
-5.88438179432653	-0.21567290399661	6.07987582057962	c
-7.70544874093410	1.97843172188750	6.65352361664097	c
-9.41669756502248	1.33698092583989	7.68576110522481	h
-6.76832622485222	3.41917723336902	7.85649587160323	h
-8.35083494196860	2.92598849600634	4.89880722478034	h
-5.05364849365182	-1.52824859965728	8.55044103371760	c
-4.16007218178567	0.60273950148141	5.19952815466515	h
-4.12463642814620	-0.17881044064766	9.86438662715473	h
-3.71349526238865	-3.09071263024879	8.16446303270218	h
-6.70789846795910	-2.35698284038909	9.54249295451019	h

Isomer **II** [**L**Si-N(Dipp)-Si**L**] of [**L**Si(NDipp)Si(**L**)]:

-0.38814507883659	-0.71396997057849	0.00205609979297	n
5.69429718461360	-0.72291867873558	-0.00008768982196	n
-3.70940900092587	4.38226286470598	-0.00226486509809	n
4.08313925837073	-2.17123178353374	3.49287549280499	n
-4.04735602207268	2.23648963428361	-3.49160733127180	n
2.51771592521663	0.45302733098434	1.51301676776061	si
-0.99358835377671	2.35635133557607	-1.51277006528054	si
6.26195461555005	-1.97176699078288	2.15109835266396	c
-5.06630205028473	4.17484319166881	-2.15289773548972	c
7.35978020758065	0.38602787269303	-1.96596832306008	c
5.67172593266748	0.96347466394957	-4.27428004642048	c
4.87872086637424	-0.79812861102665	-5.08333772466412	h
6.79461847236229	1.91593900481685	-5.76488327524325	h
4.07131157519794	2.21509508996893	-3.75109017404097	h

8.53537208945063	2.89021753504900	-1.01704777603831	c
9.46420811320229	-1.45701831407006	-2.79166485040181	c
7.03204383275313	4.21201452311022	-0.40144478967004	h
10.89289061045175	-1.77010591872915	-1.29931341208996	h
9.63871013559134	3.80713496739943	-2.54671401063360	h
10.45577202634808	-0.66752262946526	-4.46073363889794	h
9.83200044886095	2.56137926646329	0.59255630666174	h
8.66142760729206	-3.31277275070505	-3.33939515992220	h
-3.68517728386413	6.38605340965054	1.96050620780603	c
3.65255838792393	-3.05895481729011	6.11643633958893	c
-4.55899911791869	1.38668370100474	-6.11317021866692	c
-2.28806326307794	5.28508834423649	4.27300988097445	c
0.78402353510961	-2.86779043129196	6.55240358760748	c
-2.83496178144013	-0.91435673557050	-6.54656694437458	c
-3.33918215444820	3.66497844730365	5.08356642685493	h
-0.25971487496539	-4.05887435140925	5.18439047241509	h
-3.26375683097966	-2.43691135989924	-5.17633876899511	h
-2.09890759712171	6.74772517396873	5.76124060851476	h
0.29196478561080	-3.48648237317635	8.49178472685066	h
-3.08613504855703	-1.66663998483818	-8.48488138890596	h
-0.36771112822037	4.61846848699806	3.75376224813327	h
0.14013617298952	-0.88486538357470	6.32335260131571	h
-0.82156649011448	-0.37254082471415	-6.31904899878783	h
-2.21767198029340	8.73058785287480	1.01020754762051	c
5.01735134510520	-1.36879243302904	8.07212649496719	c
-3.88849080745684	3.44850545379806	-8.07365988977056	c
-6.37659409199084	7.15520047280112	2.78015375555944	c
4.46607156377219	-5.84150192727496	6.44250684579575	c
-7.33532758139742	0.55039993499174	-6.43471854827159	c
-0.29003978318441	8.18398388422764	0.40062673626260	h
4.50425112885581	0.64181817996081	7.77936490007239	h
-1.92308560286173	4.11547167195198	-7.78411230064923	h
-7.41147842522368	8.18429556924641	1.28475185406298	h
6.52857240671583	-6.09003505210328	6.20412579151196	h
-8.66819127920893	2.14425088337347	-6.19889095552080	h
-2.05034975305870	10.15826666832701	2.53721989995620	h
4.45059982233078	-1.91176382650138	10.01649521535899	h
-4.03639966565396	2.67332603172091	-10.01630057071641	h
-6.25475012059428	8.41787420785255	4.44842507861172	h
3.96629947453296	-6.50163483386961	8.36800284561065	h
-7.61795282717363	-0.23310439974070	-8.35808066526840	h
-3.19344216557808	9.63760711667245	-0.60396033256925	h
7.09704808130722	-1.55126829869113	7.94630729808055	h
-5.17542101878717	5.09252045184683	-7.95000994218446	h

-7.50134912722157	5.47461801746778	3.32686573133189	h
3.47838754714058	-7.07048966160243	5.06438851282941	h
-7.82560700354599	-0.94443288008788	-5.05258425778018	h
8.85666027941095	-2.67528922623242	3.05761674488958	c
9.83790063850292	-5.11740296300026	2.61652151858902	c
12.28160221837852	-5.75518971961217	3.43051087765887	c
13.77940095069121	-3.96524234666750	4.69633516944981	c
12.81761279387366	-1.53220414570419	5.15044893760190	c
10.37353703176705	-0.89054524521185	4.33941429965769	c
8.67544289516369	-6.51457724712774	1.60934897489198	h
13.02079830833807	-7.66537668478938	3.06706081658588	h
15.69615398876046	-4.46620751340223	5.33051659675357	h
13.97606911732460	-0.12029988432681	6.14685631025459	h
9.62439134399994	1.01303453287561	4.71145850011404	h
-7.06811178269961	5.96737008355396	-3.06320031654860	c
-9.65130505979375	5.46206946818195	-2.62386912547340	c
-11.51552177692167	7.16372359061048	-3.44252431170435	c
-10.82779982696780	9.39242708703223	-4.71116898445973	c
-8.26273819106291	9.90931810619499	-5.16314674144766	c
-6.39509419314683	8.20939316257459	-4.34761091294299	c
-10.19186209245071	3.72817781165449	-1.61426811929234	h
-13.52072239704094	6.74475617376290	-3.08067064214798	h
-12.29059947326824	10.72670678141606	-5.34914131792528	h
-7.70786017620865	11.64823524082540	-6.16149455039255	h
-4.39002765565132	8.61634509790810	-4.71822929894559	h
-1.67834072847487	-3.08907620235214	0.00368337194090	c
-0.60775269629975	-5.27455978475102	-1.21872429903128	c
-1.92485791114712	-7.58882931522526	-1.18347728973952	c
-4.26984720873459	-7.85634961936045	0.00519232526489	c
-5.32003323958527	-5.74251386083408	1.19355610203753	c
-4.09393925492592	-3.37876846304239	1.22752317175936	c
-1.07714310134222	-9.23642133135026	-2.13402533817957	h
-5.26363949011796	-9.68401703445681	0.00546458167774	h
-7.16342320938919	-5.92646260179644	2.14475539563029	h
1.94503764316454	-5.24660001814799	-2.57306454505278	c
3.84612481735423	-7.04562922155397	-1.29087878413508	c
3.23542835964148	-9.04738014096519	-1.44760416207270	h
5.73354483810214	-6.89962572842067	-2.19754655419564	h
4.05305747885157	-6.59834197022251	0.74108106117493	h
1.73512135026225	-5.91805972288488	-5.40336993402933	c
2.71035644291284	-3.30664448497874	-2.42371164920192	h
3.61387553751892	-5.79210511593695	-6.33113033397600	h
0.43309716390171	-4.63893185528690	-6.42047730514078	h
1.03942818756378	-7.87809772000967	-5.68275214805957	h

-5.45750286567898	-1.22099710799235	2.58260467685983	c
-8.00331662428228	-0.60498566556199	1.30437149640101	c
-9.35129241086022	-2.20546113529844	1.46587336206036	h
-8.90486681556044	1.05989647128944	2.21053207018730	h
-7.74419577643797	-0.19090866608512	-0.72864896772463	h
-5.90223142558483	-1.76139440704026	5.41383147818521	c
-4.24630726172149	0.47644077871870	2.43064596122576	h
-6.81813989669310	-0.11662831840962	6.34234086365710	h
-4.11894031653222	-2.15663265088459	6.42850842469488	h
-7.16709491194449	-3.41198902213398	5.69572142619780	h

[**L**Si-Si(NPh)**L**]:

-1.87654006574988	0.85228925031222	0.09033916485661	si
0.37037747654858	0.31879168660414	4.05139739598716	si
-4.52679391280515	-1.67296299126203	0.06467985828825	n
-1.37706304488697	-1.54768962578885	-2.58542195910337	n
-2.47770402719352	3.81385129850650	-0.70171814039480	n
-3.46455635361042	-2.85930164695104	-1.92022480224620	c
3.62965915409861	-0.84797084620070	2.94469348403279	n
2.71567770497106	3.03126941367050	3.86100390709477	n
4.66056477258859	1.47270797283365	3.33330440218200	c
-6.51389464039283	-2.45675475688833	1.87683826024683	c
0.10766156253167	-1.48864576131058	-4.96123663989045	c
-4.07404299798908	5.03300370105441	-2.35346717117395	c
-4.40645773711605	-5.21448864911345	-3.17286738410861	c
4.78607202706882	-3.39200931749130	3.07077273556935	c
2.55004105177011	5.81641969544213	4.13274253165674	c
7.41183779054942	2.12986562713216	3.27862522382808	c
-7.11105949724277	-0.09978949618331	3.48958421641101	c
-8.95192931960442	-3.30714192051188	0.51949355801635	c
-5.52286548433598	-4.59146411594678	3.60638102678059	c
1.21130411061820	-4.10457125563951	-5.62934251765675	c
-1.54592836295476	-0.50060220571352	-7.15697343022744	c
2.29476030739508	0.38182827464420	-4.48712069422649	c
-3.57015679097703	7.59419783121956	-3.05514050562986	c
-6.29318752991559	3.92429566387985	-3.43432911419519	c
-6.28419533651231	-5.07802507058737	-5.06446197296069	c
-3.42246244666041	-7.59672799496588	-2.48276341557507	c
6.98192176068943	-3.68650736995470	1.17387678835314	c
2.66377066167356	-5.25898355095186	2.36196798785055	c
5.71072790245582	-3.97445637810794	5.78135845462918	c
-0.20265806801702	6.39672258413157	4.89040241353068	c
3.12643657594834	7.08475678480353	1.56925570417038	c
4.34993178679360	6.79688438770313	6.21019038005003	c
8.60905417815435	2.67146790553331	0.95507905019950	c

8.85214700515094	2.21292196434601	5.52494164734448	c
-5.41974994107826	0.52993801379491	4.55612856462143	h
-8.61477925054295	-0.54270261321443	4.87836662651747	h
-7.75885609819212	1.48805821767706	2.28854013350082	h
-9.62073453749574	-1.83074477752281	-0.80771841182098	h
-10.46098272091952	-3.62656695954053	1.93776590852956	h
-8.69424328908236	-5.08987454886801	-0.54095352801092	h
-5.09028022502174	-6.32154481770089	2.50736117636838	h
-6.96078060537934	-5.09104023337661	5.04765747511532	h
-3.78921303322202	-3.97416865424371	4.61233326186592	h
2.35789569267382	-4.86407215700885	-4.04847106720586	h
2.45929171704640	-3.93272752505789	-7.30428271019627	h
-0.27827613491664	-5.49730145657659	-6.09104679453392	h
-3.06690159754657	-1.86026846584895	-7.62818981796287	h
-0.38171071557814	-0.23171026623833	-8.87935371710052	h
-2.42631825537159	1.33386012941215	-6.66065038525232	h
1.55070686864851	2.28755957399342	-4.03761006497521	h
3.50972791112856	0.52094005249880	-6.18695432021587	h
3.46880334768886	-0.25465009452714	-2.87161891423999	h
-1.88524934919715	8.50646955080402	-2.24532940189092	h
-5.14745084889527	8.92516417646002	-4.71072595022924	c
-6.76830145406283	1.96527007792385	-2.91296329193028	h
-7.87096297869607	5.27372267960362	-5.08188175499394	c
-7.07246751953025	-3.22987732037858	-5.59775859194223	h
-7.15265046999483	-7.28803790955958	-6.24749650352897	c
-1.94813199901413	-7.71461936847652	-1.02179903343383	h
-4.30731666792314	-9.80279341024016	-3.66166351112093	c
6.36422286328317	-3.19351516855191	-0.76604308819951	h
7.63995358236898	-5.67631962895270	1.15898465316494	h
8.61710411672140	-2.48176012202702	1.66757710457977	h
1.06052634190185	-5.09418139025093	3.70647788765239	h
3.36039249339375	-7.23284043269938	2.42633591187264	h
1.94723035777388	-4.85643392823861	0.43554881495262	h
7.28988152597477	-2.71421020806199	6.32954726331129	h
6.39587174381706	-5.95128123534658	5.92131557055298	h
4.14913297352255	-3.71964122535519	7.15547175267440	h
-1.51337612956369	5.73692214088691	3.38863342510196	h
-0.46265139648355	8.46189428727852	5.12166172090387	h
-0.68816350911211	5.47284524519490	6.70975993275875	h
5.09390293168207	6.70838059427199	0.95592861532532	h
2.89151053005601	9.16218284366690	1.72289894650349	h
1.78707770213233	6.36418624440117	0.12772075801002	h
4.01444271719060	5.79513159231791	8.02072597830331	h
4.00086497828621	8.83849902688344	6.52913829645059	h

6.36281669911816	6.57569008282869	5.68771573377447	h
7.49962493456885	2.61223861638967	-0.80229126200158	h
11.19121969011902	3.27830057364758	0.88116586546059	c
7.93262878193739	1.81934917063293	7.34768543029391	h
11.43616828536483	2.81050419806827	5.44463156195589	c
-4.67919123912009	10.89580399840638	-5.19944197127840	h
-7.31788557110609	7.78656339043468	-5.74757038052933	c
-9.56787879742987	4.34573348108679	-5.85879497286790	h
-6.17022076806109	-9.65417074727573	-5.54832272505140	c
-8.61324773377925	-7.15816409828499	-7.72262372059581	h
-3.53035052336219	-11.65007420899291	-3.10443731465355	h
12.09827425317072	3.70260866751674	-0.94201895957762	h
12.61118917913966	3.34466222205844	3.12377930943216	c
12.53686692216725	2.86538579481545	7.20897158397872	h
-8.55735191688631	8.84104530262082	-7.04298110050933	h
-6.85797300137783	-11.38481528953942	-6.47461678463265	h
14.63586892402433	3.81801722813337	3.06392034325928	h

Isomer **II** [**L**Si-N(Ph)-Si**L**] of [**L**Si-Si(NPh)**L**]:

-2.74820990823313	-0.20588786376188	-2.78737642880251	si
0.00000176963255	-0.00001139216599	-0.73505367077425	n
-0.00000935673786	-0.00002715188550	1.94038029257723	c
-0.00015694114704	-0.00016301392639	7.33992933064681	c
-0.00026166285418	-0.00025605024182	9.41987683781536	h
-5.14401566357129	2.39933473475338	-1.67960081728683	n
-5.33296165071171	-1.54819781713773	-0.58562666963978	n
-6.67015441381759	0.61935372279767	-0.66236388702097	c
-5.72932465726723	4.90912251701791	-2.76745199602219	c
-5.98352223977384	-4.08825078206652	0.38448875620587	c
-9.38685322041094	0.95630039211264	0.07610651887463	c
-1.48441918189292	1.74942781477208	3.33389232435198	c
-3.15685105334547	6.11106657712802	-3.43333076225421	c
-7.31541661425965	4.65041371630330	-5.20961464952624	c
-7.10529549150982	6.62915311061229	-0.85432575945567	c
-3.61963424770762	-5.74865867655531	0.00056931759122	c
-6.60743719833581	-3.96955230713539	3.23352484576172	c
-8.20607150357653	-5.27038953168006	-1.10015771165536	c
-11.36257549500893	0.45414211062443	-1.64637097369240	c
-9.99793913518668	1.80948480707908	2.53004737450295	c
-2.64403611106811	3.11311488961240	2.27941321951753	h
-1.48057260018643	1.74331818994274	5.98640287692594	c
-1.98468158006840	6.36755254636908	-1.71838021412936	h
-3.43406333637858	7.98320069865672	-4.33079705741387	h
-2.08971034927762	4.89802356563058	-4.77080166606990	h
-6.35096303645534	3.38416178248967	-6.57186398050244	h

-7.57692803501676	6.52144758502336	-6.12022589532216	h
-9.21548840476631	3.87045519791181	-4.80563229479637	h
-9.03092422205711	5.94450160246916	-0.41443702725452	h
-7.28405874320144	8.56148429524634	-1.64666503667597	h
-6.02832299325715	6.76628361076653	0.93797616353288	h
-3.08614066487877	-5.82375665221721	-2.02537472721576	h
-3.99856050064018	-7.70420068350622	0.64709140557414	h
-1.99704051136927	-4.99615093088620	1.08764252835618	h
-5.03991022416525	-3.07090187418444	4.29201374178501	h
-6.89013053395780	-5.90513440450581	3.98754301483198	h
-8.36155152780978	-2.88698130460075	3.59354130235052	h
-9.99363823057820	-4.23030055053216	-0.78816301626967	h
-8.51013472089773	-7.25184504473471	-0.48578531637761	h
-7.79147489523680	-5.28293079278334	-3.15434982312005	h
-10.90992670483978	-0.22684159609672	-3.55806796563482	h
-13.89368717114559	0.80997591801855	-0.93091501454394	c
-8.46627841026581	2.20375735211046	3.88001463112643	h
-12.53011896666939	2.15618843754517	3.24314419606686	c
-2.64445812042992	3.13270678693359	7.01269094273815	h
-14.48374651525944	1.66134226337950	1.51406710607748	c
-15.41480076566320	0.41480801528990	-2.29402988972927	h
-12.97848871641670	2.81988734669769	5.16302385493849	h
-16.46838399547134	1.93728299093275	2.07280826177371	h
2.74822910147925	0.20588772592897	-2.78735411333885	si
5.14403976818116	-2.39932533528552	-1.67956809878597	n
5.33295807537088	1.54820217906245	-0.58557955466308	n
6.67017016771656	-0.61933673855318	-0.66233287377104	c
5.72936768029001	-4.90910530297055	-2.76742637994162	c
5.98349555519623	4.08825377402365	0.38455507221436	c
9.38688653619999	-0.95625476055839	0.07608561684986	c
1.48436525936055	-1.74951761342149	3.33388526483609	c
3.15690410578527	-6.11106222274941	-3.43332074961210	c
7.31546800218944	-4.65037565869111	-5.20958135838224	c
7.10534172347569	-6.62913551776434	-0.85430206435625	c
3.61959845771645	5.74864767407536	0.00063106996543	c
6.60739525465396	3.96954402498664	3.23359414961090	c
8.20604522850709	5.27041561427175	-1.10007221469913	c
11.36257108538778	-0.45407410066233	-1.64642861307124	c
9.99802715794821	-1.80944182773928	2.53001188775209	c
2.64408593172943	-3.11311243295864	2.27940099858813	h
1.48037345758308	-1.74354145724903	5.98639602045877	c
1.98472896819900	-6.36756206249379	-1.71837618659817	h
3.43413142616186	-7.98319108756328	-4.33079336195317	h
2.08976163768568	-4.89802032243029	-4.77079126288644	h

6.35101148832007	-3.38412453902850	-6.57182936761760	h
7.57699582130967	-6.52140391795381	-6.12019938450412	h
9.21553287352545	-3.87040577506163	-4.80558787895097	h
9.03096430873791	-5.94447373792175	-0.41440242391525	h
7.28412051558038	-8.56146210520623	-1.64664907493673	h
6.02836276993567	-6.76628074521729	0.93799482782189	h
3.08611648881952	5.82375393718463	-2.02531574273383	h
3.99850718264577	7.70418844142558	0.64716715397103	h
1.99700341669768	4.99612217581181	1.08769004727773	h
5.03986884941992	3.07087659604878	4.29206943151288	h
6.89007070665194	5.90512399047813	3.98762454196515	h
8.36151526177973	2.88698358943086	3.59361439948092	h
9.99361722143000	4.23033684490906	-0.78807325739670	h
8.51009162135014	7.25186966253874	-0.48568685042908	h
7.79146026609554	5.28296576661770	-3.15426663547211	h
10.90987909068390	0.22690249181398	-3.55811790865524	h
13.89369977660052	-0.80988728613341	-0.93102251096717	c
8.46639670417753	-2.20371228385044	3.88001404757391	h
12.53022304324724	-2.15614276341938	3.24305278668611	c
2.64425154216010	-3.13294074008721	7.01267802418724	h
14.48381266178595	-1.66127945702728	1.51393767026676	c
15.41478493382253	-0.41467053453349	-2.29415494517403	h
12.97863532918440	-2.81985335591707	5.16291845764332	h
16.46846199836477	-1.93723496378282	2.07262941715903	h

[{**L**Si(NDipp)Si(**L**)}-CuI] **2d:**

0.00002494206420	-0.00009009503830	-5.05352720938792	cu
0.00005780949525	-0.00015170996369	-9.81087305249988	i
-2.50995537504088	-0.24819333607556	-1.50659047006341	si
0.00000138524091	0.00001445549392	0.87269024410055	n
-0.00000250591467	-0.00000475736409	3.59360686078336	c
0.00058273787751	0.00036615804519	9.02887919173791	c
0.00088269007326	0.00056143609619	11.10876733849036	h
-5.05840519565085	2.35235755910176	-1.77015881707120	n
-5.60879086985593	-1.39483382758027	-0.17965508439149	n
-6.85035120654589	0.64191900076620	-1.14926893534888	c
-5.22881560011859	4.67879333932153	-3.35345369083841	c
-6.55828116914959	-3.90508438267375	0.63637495167167	c
-9.61973955952497	0.79209027131491	-1.71886399534716	c
-1.32893389285914	1.91027152693315	5.00772202003208	c
-2.55430290873937	5.83439290226663	-3.40974426752491	c
-6.02557926870154	4.05482605011313	-6.09114375514467	c
-7.07364567720274	6.60587948135043	-2.17616587311386	c
-4.27143909563017	-5.27726459259860	1.79463275585726	c
-8.62649408868515	-3.62911879617368	2.67301693012841	c

-7.55115458634462	-5.45907443145797	-1.62944880661359	c
-10.58439853668238	-0.19660465707786	-4.00449539532749	c
-11.30273747292826	1.92739302630592	0.01333463857933	c
-2.88321728606450	4.03181625577554	3.81357882389273	c
-1.28676910758484	1.86082736452657	7.67069305390949	c
-1.90123321976636	6.35365525465567	-1.48854828906596	h
-2.53957533119822	7.55699319449567	-4.59835580042568	h
-1.17821815665169	4.47796414961846	-4.23901763770752	h
-4.72708257114504	2.63811541206960	-6.92798714206733	h
-5.93215672113712	5.78350784250735	-7.27214459828926	h
-7.98644863168633	3.33435606015189	-6.18325145200672	h
-9.04128138579085	5.89837630747177	-2.15543572625946	h
-7.04798275847849	8.37075709601647	-3.30466123481643	h
-6.51746115736092	7.08673331370365	-0.21552799039608	h
-2.73942380574544	-5.48573676997391	0.37770077120629	h
-4.81965355046629	-7.19408913838500	2.43386242805416	h
-3.51391750264253	-4.21709837321552	3.43220911830763	h
-7.92170818859236	-2.52091439530386	4.30474542660010	h
-9.18064924646733	-5.52498076226250	3.37241550698439	h
-10.34581815966584	-2.70199624768997	1.92735801910748	h
-9.30104595304132	-4.63771307963869	-2.42660636006031	h
-7.98978088901413	-7.41695990253948	-1.02282463517455	h
-6.10669583663762	-5.54997137551638	-3.14435055227182	h
-9.28966199534373	-1.07029206840057	-5.37618671049785	h
-13.17950085281435	-0.05164078972318	-4.53921591375005	c
-10.56567170041497	2.70225415805511	1.79493301109076	h
-13.89747107270219	2.06339769122217	-0.52656271424391	c
-2.74600045885806	3.83988716775512	1.73805854988182	h
-1.91923598619505	6.69056397145639	4.52467064803646	c
-5.69733283547140	3.77885934151819	4.53622426166298	c
-2.30976457056106	3.34626587001220	8.70933667012591	h
-14.84187196544056	1.07511778843776	-2.80228742371492	c
-13.90316871186727	-0.82263327867800	-6.33037986337204	h
-15.18734690852997	2.95045900223183	0.84296710291617	h
-2.08814955688502	7.05172149036662	6.58423869735950	h
-3.04420420471629	8.16658549904294	3.54541189611973	h
0.08581228004285	6.96347111527430	4.00129414985059	h
-6.44171361265751	1.89046743590274	4.02848459748538	h
-6.85325522876891	5.23069408387274	3.55999931805176	h
-5.98254812527633	4.03885297938505	6.59798594598247	h
-16.87548262257273	1.18561921949648	-3.22497587010840	h
2.50997067997963	0.24815661611328	-1.50658244407002	si
5.05842879152107	-2.35237715396276	-1.77008078067134	n
5.60879557308151	1.39484746577220	-0.17967909264369	n

6.85037267092438	-0.64192789769381	-1.14924767262609	c
5.22888892687210	-4.67873354339387	-3.35345736256410	c
6.55823484973336	3.90505973393941	0.63654430241703	c
9.61974909121157	-0.79215261363530	-1.71888380100703	c
1.32910321821012	-1.91017008949987	5.00770433349374	c
2.55439585299054	-5.83438283612086	-3.40980763945886	c
6.02566535557265	-4.05459421153740	-6.09110785278167	c
7.07377594901492	-6.60581238933938	-2.17624953222015	c
4.27131333133829	5.27712350927805	1.79477944397601	c
8.62634489029799	3.62900911664228	2.67328059899490	c
7.55117726058675	5.45923972038149	-1.62912063645039	c
10.58437914787271	0.19654198473318	-4.00452827616000	c
11.30275398264045	-1.92753980195835	0.01325068883434	c
2.88285515121014	-4.03207338537378	3.81354701430755	c
1.28756378709186	-1.86033443988918	7.67067331243249	c
1.90132643021010	-6.35373044912041	-1.48863483744038	h
2.53971002125732	-7.55693915583218	-4.59848260844181	h
1.17828490051516	-4.47795365477770	-4.23903662957282	h
4.72713503383533	-2.63787378099150	-6.92788285921241	h
5.93231822371570	-5.78320660122067	-7.27221416179171	h
7.98651217424051	-3.33404700425909	-6.18313793359885	h
9.04139520748092	-5.89825699774411	-2.15547670900026	h
7.04816339520386	-8.37062920301923	-3.30483973384181	h
6.51759805646373	-7.08679043367398	-0.21564022743442	h
2.73936052424869	5.48565344271459	0.37778811630950	h
4.81946245577366	7.19391916416608	2.43415204002042	h
3.51373579567304	4.21684549206786	3.43225758988138	h
7.92148888895616	2.52069831023075	4.30490676420060	h
9.18043243082135	5.52483931724046	3.37281957872221	h
10.34572211345017	2.70195962154843	1.92765935487701	h
9.30113426752295	4.63799261009681	-2.42624566774223	h
7.98971330266556	7.41709548696985	-1.02233609475319	h
6.10679106504666	5.55020170463862	-3.14408794462334	h
9.28956913285258	1.06985997104452	-5.37638522051115	h
13.17950686666563	0.05178192200070	-4.53918046395693	c
10.56569335778464	-2.70252366217298	1.79479846112238	h
13.89748360901486	-2.06352686083534	-0.52667043581895	c
2.74523068103112	-3.84056136598498	1.73801015340048	h
1.91878342605629	-6.69053162554018	4.52556070097994	c
5.69709277936013	-3.77925876366486	4.53572320821427	c
2.31080613590748	-3.34562018551576	8.70929763667847	h
14.84187894262362	-1.07510637894502	-2.80233648542344	c
13.90322109017441	0.82320576576717	-6.33013996062502	h
15.18735610702373	-2.95071420228837	0.84278091717124	h

2.08811913231869	-7.05115282922302	6.58519189108868	h
3.04339970050663	-8.16691373456449	3.54644577993551	h
-0.08640538447238	-6.96338923838064	4.00270007577806	h
6.44159605972759	-1.89105265597491	4.02747325416020	h
6.85271790900093	-5.23142732931237	3.55964701775861	h
5.98258091963825	-4.03883794110422	6.59750521122252	h
16.87548177054828	-1.18562604359569	-3.22505774774522	h

CuI

0.00000000000000	0.00000000000000	-2.24840890991765	cu
0.00000000000000	0.00000000000000	2.24840890991766	i

ITMe

0.00069533993475	1.71514734823750	-2.01569242616217	n
0.00069533993475	1.71514734823750	2.01569242616217	n
-0.00035372615631	-0.84234207798607	-1.30409556730379	c
-0.00035372615631	-0.84234207798607	1.30409556730379	c
0.00136206525761	3.35471186336236	0.00000000000000	c
-0.00120103967763	-2.97825298086616	-3.15169677859427	c
-1.68888707060287	-2.94698556248464	-4.40222528731554	h
-0.00207479148980	-4.81710232080175	-2.15333003465796	h
1.68665027372211	-2.94849315918008	-4.40204292468562	h
-0.00120103967763	-2.97825298086616	3.15169677859427	c
-1.68888707060287	-2.94698556248464	4.40222528731554	h
1.68665027372211	-2.94849315918008	4.40204292468562	h
-0.00207479148980	-4.81710232080175	2.15333003465796	h
0.00104695887548	2.59321008486440	-4.61608726676354	c
0.00180096424193	4.68025375277365	-4.56379822754429	h
-1.70065020907068	1.93421880783110	-5.65198906534179	h
1.70229226759427	1.93299017593093	-5.65194924790588	h
0.00104695887548	2.59321008486440	4.61608726676354	c
0.00180096424193	4.68025375277365	4.56379822754429	h
1.70229226759427	1.93299017593093	5.65194924790588	h
-1.70065020907068	1.93421880783110	5.65198906534179	h

CuI-ITMe

0.00005068219159	0.90206800844562	-2.04087955178219	n
0.00005068219159	0.90206800844562	2.04087955178219	n
-0.00011629959583	-1.63779591554542	-1.30638390771490	c
-0.00011629959583	-1.63779591554542	1.30638390771490	c
0.00014880514759	2.49607298406700	0.00000000000000	c
-0.00023627863206	-3.76966202693879	-3.15550308032476	c
-1.68953033234043	-3.73205513426768	-4.40088555958428	h
-0.00044006171140	-5.60551726324921	-2.15627784200530	h
1.68916201700869	-3.73236253003446	-4.40075569786910	h
-0.00023627863206	-3.76966202693879	3.15550308032476	c
-1.68953033234043	-3.73205513426768	4.40088555958428	h

1.68916201700869	-3.73236253003446	4.40075569786910	h
-0.00044006171140	-5.60551726324921	2.15627784200530	h
0.00009472714168	1.79496096454785	-4.64293109885947	c
0.00035646618108	3.88579343439783	-4.59027198659734	h
-1.70550064288055	1.13612986585808	-5.66549829180522	h
1.70547343319804	1.13570013562562	-5.66557982593188	h
0.00009472714168	1.79496096454785	4.64293109885947	c
0.00035646618108	3.88579343439783	4.59027198659734	h
1.70547343319804	1.13570013562562	5.66557982593188	h
-1.70550064288055	1.13612986585808	5.66549829180522	h
0.00039363510516	6.06297113768793	0.00000000000000	cu
0.00083013862566	10.68643680056626	0.00000000000000	i

TS1

-0.84671140445324	-1.84051876035274	-0.47487911118137	n
5.25515690819305	-1.58515241272642	-0.20056408438267	n
-1.42972257811961	5.37011425934404	-0.71742281130773	n
3.41404054572360	-1.80472177794440	3.50252610431936	n
-3.81317717052649	2.97102251413982	-3.07393915015468	n
1.77407860355774	-0.39595603184945	0.55775873156651	si
-0.15681065012847	2.43715130091409	-2.46397312676738	si
5.61486414685610	-2.14308332265812	2.25853697468997	c
-3.70403406685881	5.22489733227338	-1.87578610698620	c
7.15623287401122	-0.79370446153412	-2.11968482576837	c
5.83310641562004	-0.81886460728725	-4.71632647215091	c
5.25477577896104	-2.75990125635484	-5.24127328798823	h
7.14532800274850	-0.10979248329375	-6.18735187501312	h
4.12861410462025	0.40348893020649	-4.72088380078694	h
8.06066508780813	1.92413938739295	-1.53879876064532	c
9.44497481119195	-2.60001242295705	-2.25547159119312	c
6.43700548163611	3.24718346370478	-1.50198008158170	h
10.69184706895732	-2.46775144790855	-0.58499416549906	h
9.40346617298500	2.58483559246249	-3.00733641776200	h
10.59467426996383	-2.11378238370773	-3.93892126161046	h
9.03969518863634	2.01934984057040	0.31092094649362	h
8.81741708091390	-4.58750337570937	-2.46032360473559	h
-0.50344530912524	7.06009092717302	1.31174546700904	c
2.50262568544017	-2.73627809728373	5.97866553489862	c
-5.25875312637805	2.29296026355856	-5.39666590554392	c
2.40780230693474	6.90146562687392	1.28225733708046	c
0.42361087443339	-0.88401228837481	6.83976219806642	c
-4.77706433598758	-0.54243049708945	-5.85027947539502	c
3.03520889447310	4.91668500613405	1.52367821255275	h
-1.07647345402800	-0.71196604808779	5.39189899209035	h
-5.51069794437230	-1.70526669833136	-4.27459412614710	h

3.21114130445222	8.04773013237567	2.84038563280422	h
-0.46242582571937	-1.55373787585815	8.61460573847785	h
-5.70453645222263	-1.16023368173536	-7.62383821270102	h
3.17287964010567	7.62442403735615	-0.52926352489494	h
1.22924184189652	1.01913616581037	7.18253435912508	h
-2.72346376749651	-0.92568098262964	-6.01690356718915	h
-1.26097934179141	9.85474484720626	0.94542799215365	c
4.55246010151262	-2.80604448452553	8.05268578516166	c
-4.26270124731089	3.81346297565422	-7.68797919421370	c
-1.52260905770439	6.09952023385378	3.87598578283751	c
1.39155509588239	-5.41387025879153	5.60827573233754	c
-8.12942214969597	2.72642971113573	-5.11037922502527	c
-0.68361228837660	10.55587566273052	-0.94138378468360	h
5.48398683827551	-0.94399102870692	8.27222905744394	h
-2.20031630153005	3.52309755009701	-7.91784555326212	h
-3.61447924549637	6.20688900522207	3.93053406254138	h
2.88916150093571	-6.75493555646418	5.01463308935917	h
-8.62967917218718	4.75241888059996	-4.99812874973432	h
-0.28159850616014	11.01712145899284	2.38889346531475	h
3.63837357416938	-3.28409224986986	9.87692186952571	h
-5.21902126543524	3.19457988160355	-9.44902792008350	h
-0.77508680474828	7.25625331238192	5.45733102701327	h
0.56155436877140	-6.12857506328854	7.39595480169545	h
-9.10936417284890	1.92435058072861	-6.78049405514835	h
-3.31370439677259	10.17173697625649	1.16819776084383	h
6.02666836127463	-4.24157318811569	7.69257675260189	h
-4.62321781664524	5.86378220112214	-7.45912356456504	h
-0.95880375921804	4.10731448051697	4.18242517187703	h
-0.09931756413615	-5.41494641089573	4.13800931587625	h
-8.87727816517014	1.76199831627152	-3.41035159230506	h
8.06200593055733	-2.92410729952490	3.44953645166563	c
8.83538392696076	-5.47438823501351	3.33587518030656	c
11.11564225638251	-6.23149200393421	4.45974300929703	c
12.65206642780589	-4.45048678646129	5.69362067695899	c
11.89323662039150	-1.90791281585679	5.80901207819019	c
9.60520354421896	-1.14703627485421	4.70248783664583	c
7.63989652930670	-6.86378965547689	2.35391765078687	h
11.69883139116972	-8.22591796104882	4.36531831576198	h
14.44341600962882	-5.04506568524695	6.56780792822241	h
13.08837880069413	-0.50553231408560	6.77472510086062	h
9.00246899397665	0.84011348902614	4.81565895692072	h
-5.66820003478377	7.25914371059733	-1.96729565375984	c
-7.79272183861588	7.11066030930099	-0.35928381639704	c
-9.65153458971175	9.00367063373427	-0.41208851103157	c

-9.42079096214145	11.05453150199773	-2.08290850359354	c
-7.31613777674302	11.21023014739497	-3.69654109695248	c
-5.44539810376486	9.33122780430093	-3.63429941181479	c
-7.98461416535406	5.48782831355035	0.92635512038009	h
-11.29628711014412	8.86857054010541	0.85434531586501	h
-10.88318683874014	12.53320905200958	-2.12984713143250	h
-7.12555291793563	12.81232784127435	-5.01003019421451	h
-3.78662870118026	9.47234010745982	-4.88007506027697	h
-2.31435027278349	-3.98483254921558	-0.76759013232057	c
-1.67245301423866	-5.95942126549551	-2.53284185011286	c
-3.29797984913167	-8.03851128872421	-2.84666131013465	c
-5.55695298437434	-8.26470403654780	-1.48225171996852	c
-6.18906416358242	-6.36962098585570	0.25656118615352	c
-4.63150708243758	-4.25151458633917	0.65242771391637	c
-2.77906740092446	-9.53039665448868	-4.20434398818584	h
-6.80673800365592	-9.90384172077401	-1.76495090710438	h
-7.95410407758074	-6.54334700913248	1.34987693497513	h
0.79373292315561	-5.85214674894359	-4.01730994735046	c
2.79431701672987	-7.60433430891717	-2.81941925372983	c
2.17631994001780	-9.60915423221879	-2.89293485137702	h
4.63282672904601	-7.46781726974215	-3.82523461083811	h
3.11823535541867	-7.11377385954938	-0.80862255265160	h
0.47515801532739	-6.43096077812596	-6.85157026804177	c
1.49110131873553	-3.88515130893848	-3.84239631473789	h
2.28295774279043	-6.13537363945463	-7.87593947208936	h
-0.97059015854670	-5.19523398303039	-7.72994101454990	h
-0.10119726487805	-8.41981318074500	-7.19294571736124	h
-5.42998702335398	-2.26935960727122	2.59233916904458	c
-7.67388869386594	-0.66673035722488	1.65753775148626	c
-9.36324498419537	-1.85464224292808	1.28093287736281	h
-8.21895403103945	0.76873682702717	3.09178206553825	h
-7.17259771766708	0.33717259841657	-0.10745895044739	h
-6.01629768800472	-3.42323726798911	5.20173770025215	c
-3.79432370050626	-0.97101306865633	2.78527242713958	h
-6.38449118912472	-1.91734748563679	6.61682733601482	h
-4.43163103540974	-4.59763843111800	5.90592049539907	h
-7.72164424741838	-4.64504417799065	5.14624156519917	h

LM1

-1.59683295487326	-2.87381131610388	-0.03636498381890	n
4.34911459030814	-2.84235929702968	0.06689781293148	n
0.30027314585669	5.34549257314315	0.04597812705487	n
2.41880106707632	-1.49660046659952	3.47738355158210	n
-0.91460159871291	3.72060816670151	-3.50506958398013	n
0.97976087407764	-1.18288152882142	0.20339061510125	si

2.15049486197923	2.86615884926974	-1.82032742585727	si
4.56731524222940	-2.56502861523627	2.57592321927024	c
-1.42353030783715	5.59131733111768	-1.84417140746089	c
6.36795867820879	-3.28677672719483	-1.84946297302793	c
5.18921548714700	-2.80324159348762	-4.46814374876184	c
3.52157671512490	-4.02314312338459	-4.79546235679702	h
6.59582600706181	-3.20564097431715	-5.96612968364616	h
4.61084292092503	-0.79596265241255	-4.65834023376188	h
8.61661284289199	-1.45362122154281	-1.51717060237913	c
7.27589319421701	-6.05891112850816	-1.72441063004877	c
7.94412590692823	0.53231675905119	-1.53026563965864	h
8.28988376732307	-6.46412715990498	0.05920219079512	h
9.95904403044032	-1.68900137246410	-3.10946165846010	h
8.59383350137344	-6.44986668596751	-3.30635622455647	h
9.67157922485213	-1.80524512489489	0.25300301886746	h
5.65459848506943	-7.37486694977717	-1.88473608128189	h
1.11064966003542	7.16954096597865	2.00970263964505	c
1.23082275969213	-1.76264236766364	6.01679128094952	c
-2.14230958950813	2.95821306524668	-5.90365788385286	c
3.34823237871242	5.92011899143249	3.39922004913603	c
-0.97764897609790	0.12999937456133	6.11164151423500	c
-0.95351670270233	0.41365958744502	-6.67007397965387	c
2.80058486325786	4.05335569403183	4.17318745966138	h
-2.27854665295493	-0.16418464134272	4.50288897158446	h
-1.32395647571857	-1.05207563320225	-5.22045448396050	h
4.00369579497326	7.13244078882471	4.97574133408390	h
-2.05857505049174	-0.11719879585031	7.88733100251909	h
-1.77247680082268	-0.25069713991949	-8.47818945382897	h
4.95781631253461	5.62882060515118	2.08579334280843	h
-0.27311305515229	2.10035026663352	6.05101042114037	h
1.11650289034512	0.61095911350867	-6.94100433011221	h
2.03393043587438	9.67541829583095	0.82334115540432	c
3.04928177509804	-1.15064197145004	8.21329704504665	c
-1.60276019073510	4.92248496550090	-8.00041959885490	c
-1.04101519825413	7.68757959732285	3.91149172808625	c
0.21246657197711	-4.48777571287276	6.28492837161853	c
-5.01209277703368	2.59305680379310	-5.55724696155564	c
3.52458269058059	9.30285058176138	-0.60207881038239	h
3.94420893864281	0.72655048708769	7.96312962129581	h
0.45700045173113	5.26579103456052	-8.17772489719362	h
-2.63685095835578	8.69941272415609	3.01559733718090	h
1.78580274979085	-5.87246003743655	6.28913332672366	h
-5.97029894353474	4.37793079605574	-5.03552183422593	h
2.84070567761181	10.92602725556097	2.30027697226256	h

1.95272258504783	-1.10423708670279	9.99874878243101	h
-2.30941868224876	4.21961733879470	-9.84424962567410	h
-0.33757237027003	8.87633372927261	5.48792921767406	h
-0.84319404948544	-4.71185554413515	8.08195362963472	h
-5.85680571228078	1.91995200646377	-7.35246876073794	h
0.47138458862504	10.71420797995907	-0.10169564644876	h
4.55834066605164	-2.57655982220033	8.44217415139449	h
-2.55374441653976	6.74491133614781	-7.61272840881305	h
-1.76889735540243	5.89598953915179	4.71348853382955	h
-1.05976832370999	-4.93521884657058	4.68356565427246	h
-5.40263043048884	1.14797256383917	-4.09273832390108	h
6.81292292296167	-3.23406602507053	4.16090459517774	c
7.29909097834054	-5.77661021724669	4.80500637678614	c
9.36957980716783	-6.39385758681572	6.34761791653058	c
10.98403690662459	-4.48596976750190	7.24194765339135	c
10.51490662003516	-1.95122725046941	6.59778323424683	c
8.43572609436311	-1.32397722171194	5.07793121854308	c
6.03281528237752	-7.27238049669981	4.11150047155074	h
9.72090863879335	-8.38106077831854	6.85140421156152	h
12.60985937981131	-4.97324083876731	8.44430698894970	h
11.77499754695923	-0.44844498383063	7.29100436898134	h
8.06002631195783	0.66054486773294	4.58643302157782	h
-3.37162769261965	7.62571399351859	-2.10390795313887	c
-5.67400821043418	7.45431965912760	-0.76284031692610	c
-7.51437813579035	9.35124199807776	-0.99853392956924	c
-7.07831050468119	11.44873769858959	-2.56714043816390	c
-4.79171649721898	11.64089706060449	-3.90307381729909	c
-2.95073696295342	9.74281599088212	-3.67686420547733	c
-6.03005167347549	5.80214550328848	0.44620910008021	h
-9.30493558349480	9.18179912637223	0.04623611451557	h
-8.52257252422320	12.93447244640764	-2.75073557415373	h
-4.43403122715672	13.28189229705912	-5.13061374471280	h
-1.15770758444889	9.90929082757091	-4.71554629542622	h
-3.64713568228735	-3.82404126049971	-1.28991277480662	c
-3.38308926222020	-5.85120722909640	-3.10869274856065	c
-5.51483392645107	-6.77217532951340	-4.39564171322574	c
-7.94521570440847	-5.81395249751682	-3.94482491686802	c
-8.24199713988070	-3.92139648578990	-2.11507522963850	c
-6.17651188648783	-2.91815639321204	-0.77589495703904	c
-5.27473243877185	-8.30267238105345	-5.78564598264067	h
-9.58872376187518	-6.55500798334123	-4.98262660147833	h
-10.15354225761122	-3.20448609691739	-1.70770009191591	h
-0.80525694141840	-7.07987249392776	-3.48003230089685	c
-0.33212512317660	-9.07813540218826	-1.40566050178169	c

-1.72192494820087	-10.64342589278684	-1.55374234013663	h
1.59041380667391	-9.91112433606139	-1.56443654506730	h
-0.51928148854250	-8.22451787662910	0.49670378008994	h
-0.36125575564124	-8.20115894603555	-6.12203560002734	c
0.61437796180348	-5.56464697908384	-3.17556976955493	h
1.62366007125637	-8.85120038661744	-6.32345168112042	h
-0.73887737533311	-6.80004440376879	-7.63485198124355	h
-1.58090782794487	-9.86930565950369	-6.48785707802148	h
-6.61986660119393	-1.02139010056993	1.34922901800588	c
-8.82432258815125	0.81961264062296	0.89690610421077	c
-10.68111634369470	-0.15778031881528	0.89846110773214	h
-8.90716677097084	2.25432344120776	2.42725657902918	h
-8.64635038438285	1.82314687640588	-0.93326634732315	h
-7.00276162066467	-2.43166406295187	3.87712164337063	c
-4.85452718299053	0.10855746052632	1.50717856912257	h
-7.18444113216555	-1.09854880847189	5.49003677505553	h
-5.40373933784785	-3.72204941355066	4.27405505005348	h
-8.74755594574772	-3.59518047054667	3.80339339669578	h

TS2

-2.12790918122192	-2.65589944000990	0.13219603932275	n
3.79935766922847	-2.74304555335898	0.10429852011934	n
-1.31117840494200	5.44189347582378	0.44559653128475	n
2.06759350694985	-2.06161797015716	3.80100785417125	n
1.16002235597085	4.89912605986451	-2.78767704048026	n
0.46533302057471	-1.10272362333105	0.66250796521665	si
1.78094680007953	3.51649065342138	0.56667974356230	si
4.16291988836333	-2.89016539338849	2.61239220373780	c
-0.87929928917480	6.19379142988003	-1.95634209795439	c
5.59786588818373	-3.24309890341243	-1.98960567483690	c
4.16377344707830	-2.63836371028072	-4.45152942033497	c
2.58157985198095	-3.97178673538725	-4.74017424066667	h
5.44608061974158	-2.77288200468657	-6.10094544431630	h
3.35937926924998	-0.71143776750337	-4.40188172510365	h
8.01217596825952	-1.60850119515907	-1.78659609904779	c
6.35077591234379	-6.06736656075539	-2.06322994884783	c
7.54387204481958	0.40282442824880	-1.45162470496197	h
7.54928988809035	-6.59863796699193	-0.43533351813453	h
9.12140524887286	-1.73891568528286	-3.55996464263935	h
7.44551351261736	-6.45772347069047	-3.80755350621199	h
9.24326030751189	-2.24812682028686	-0.22266808662364	h
4.65214484006872	-7.28909251953465	-2.07542918463573	h
-3.03911548924983	6.54130984746117	2.35980471249608	c
1.18287350997051	-2.67948180695253	6.39673578104787	c
2.60487362603191	5.20688199085202	-5.17310199577002	c

-2.82387414016474	4.85447555454827	4.70950692782120	c
-1.12297300653117	-1.00421999616921	6.94434018257670	c
5.31312734233655	4.28533552288747	-4.63951754183136	c
-3.43433435787533	2.90465197983354	4.27336599497988	h
-2.55199855252947	-1.18994635654846	5.42915630754706	h
5.32175405022658	2.33739291749966	-3.89506493194391	h
-4.02339123456321	5.58807838266259	6.26057746660180	h
-2.00738184392020	-1.56971846105913	8.75584029336315	h
6.45024034775997	4.30109620520697	-6.39818186602731	h
-0.84167333400890	4.80481542176373	5.39447035662150	h
-0.55405822596388	1.00003947046399	7.09838550089789	h
6.25094537717216	5.51624248131641	-3.22815460432994	h
-2.23094013517542	9.25516658541899	3.08987963556879	c
3.18367112885108	-2.15194191395926	8.45647843185709	c
2.82535412483564	7.98051095186441	-6.07278934023053	c
-5.80202407579723	6.52383609501299	1.43110447503227	c
0.36616210932963	-5.48503196416085	6.46274963844923	c
1.36484567135306	3.61061582398159	-7.28207932889435	c
-0.20357525107644	9.30858700758373	3.61704587766452	h
3.93645127594520	-0.20493245293924	8.28513145284888	h
3.50679187968461	9.22201940653056	-4.52933620471779	h
-6.10333526957999	7.81561005747883	-0.18400570388946	h
1.99280242247972	-6.75540960861726	6.10704358902708	h
-0.54288292186916	4.34451504324522	-7.73562882552380	h
-3.35110483942392	9.91870445765335	4.73287488400210	h
2.27967256406143	-2.32975736329682	10.33918334539573	h
4.22737123724733	8.07197412636567	-7.62800875283558	h
-7.07294800691008	7.13787673821253	2.98029520543734	h
-0.42877330828728	-5.97636438586147	8.33936954555566	h
2.51213370646257	3.67866524345693	-9.03540228356863	h
-2.55603741667735	10.60582159598480	1.52881823025923	h
4.78769890738518	-3.48823180274583	8.40337668205440	h
1.02944356537788	8.74885997597183	-6.80697820086570	h
-6.37011126501251	4.59673890840871	0.85256238671571	h
-1.08986737217373	-5.85001279948422	5.00324390535195	h
1.16082671518037	1.61114470459354	-6.70282573816067	h
6.55872226413988	-3.66540832074249	3.90757857262169	c
7.14261690050005	-6.22512518085682	4.37244582054828	c
9.38709156834208	-6.87638227896617	5.63266536795166	c
11.07215326069874	-4.98457735533196	6.42543427555131	c
10.50218092860271	-2.43129514191655	5.96112079144263	c
8.25617174248100	-1.77054341338817	4.71786791678653	c
5.82779456252225	-7.71293739447069	3.75702514815798	h
9.81901777935957	-8.87846596780389	5.99519362795837	h

12.83160914488217	-5.49934150777899	7.40813480302213	h
11.81411383648476	-0.93954755093683	6.57793545730462	h
7.79543481210458	0.22661254782179	4.36591414394276	h
-2.35725393614406	8.12242698781031	-3.41255389013354	c
-4.28597312888272	7.29845078905493	-5.06401102296772	c
-5.69085297412004	9.05430324936702	-6.47509611304622	c
-5.18521884797895	11.65135361006580	-6.25857393262420	c
-3.26640361753611	12.48804408191011	-4.62367412734924	c
-1.86251344563240	10.73801864394161	-3.20978713655212	c
-4.69803403789130	5.26613000006827	-5.21896021455847	h
-7.19340206510249	8.38526928716667	-7.74807096233412	h
-6.28420344393115	13.02644022313230	-7.36679105921673	h
-2.84905576557316	14.51919663021342	-4.45611575371959	h
-0.33096275587681	11.40228333477726	-1.97247974592003	h
-4.08683741524105	-4.02168687613976	-0.84396814162333	c
-3.68221392045266	-6.27239624895182	-2.34656717522226	c
-5.74737126916491	-7.59628577752383	-3.36513995181913	c
-8.24921028206945	-6.83839207093101	-2.94642104368514	c
-8.67668342208475	-4.71901186033831	-1.41650839293533	c
-6.68600886592303	-3.31550823870446	-0.35707804069184	c
-5.38405812808829	-9.29833298400255	-4.51054684336492	h
-9.83844313483769	-7.89674255322417	-3.77217706312987	h
-10.63775159289331	-4.14078726413586	-1.02287674570465	h
-1.03188101337276	-7.34013030119353	-2.71106166808071	c
-0.56338367776261	-9.56501619802942	-0.88157177960580	c
-1.88080378288594	-11.15042105450420	-1.27587554401047	h
1.39415064728536	-10.31109200281909	-1.05331593511945	h
-0.86322550449631	-8.97902024117110	1.10711520343060	h
-0.45570570792376	-8.11746404158467	-5.45568285810653	c
0.29560679985703	-5.81177796157460	-2.17086034764876	h
1.54789567120896	-8.70731445139432	-5.66947290908867	h
-0.81688216400374	-6.54465410438998	-6.79381008781096	h
-1.63117024030873	-9.74163077578520	-6.07398585016826	h
-7.28486047066182	-1.15764071789057	1.45439353077142	c
-9.55587755918931	0.46826406495549	0.64565769528128	c
-11.34672783191486	-0.62485331651870	0.66544838587532	h
-9.82827321813644	2.07954226861618	1.96069291997299	h
-9.31213710682244	1.23652470070953	-1.28901991252185	h
-7.70133906604722	-2.19909948731371	4.15035364725230	c
-5.57271841791932	0.06072821315215	1.49216594906052	h
-7.92499903145246	-0.65460261789183	5.55682644518522	h
-6.10078851902926	-3.40571773083402	4.75426873828510	h
-9.43101281590660	-3.38653290267517	4.22007402788337	h

6. References

[1] a) C.-W. So, H. W. Roesky, J. Magull, R. B. Oswald, *Angew. Chem. Int. Ed.* **2006**, *45*, 3948-3950; b) S. S. Sen, H. W. Roesky, D. Stern, J. Henn, D. Stalke, *J. Am. Chem. Soc.* **2010**, *132*, 1123-1126.

[2] W. A. Merrill, J. Steiner, A. Betzer, I. Nowik, R. Herber, P. P. Power, *Dalton Trans.* **2008**, 5905-5910.

[3] T. Tsuda, T. Yazawa, K. Watanabe, T. Fujii, T. Saegusa, *J. Org. Chem.* **1981**, *46*, 192-194.

[4] a) G. Sheldrick, *Acta Crystallogr., Sect. A* **2008**, *64*, 112-122; b) G. Sheldrick, *Acta Crystallogr., Sect. C* **2015**, *71*, 3-8.

[5] O. V. Dolomanov, L. J. Bourhis, R. J. Gildea, J. A. K. Howard, H. Puschmann, *J. Appl. Cryst.* **2009**, *42*, 339-341.

[6] a) J. P. Perdew, *Phys. Rev. B* **1986**, *34*, 7406-7406; b) J. P. Perdew, *Phys. Rev. B* **1986**, *33*, 8822-8824; c) A. D. Becke, *Phys. Rev. A* **1988**, *38*, 3098-3100.

[7] TURBOMOLE V7.6 2021, a development of University of Karlsruhe and Forschungszentrum Karlsruhe GmbH, 1989-2007, TURBOMOLE GmbH, since 2007; available from https://www.turbomole.org.

[8] K. Reiter, F. Mack and F. Weigend, *J. Chem. Theory Comput.* **2018**, *14*, 191-297.

[9] a) R. Heinzmann, R. Ahlrichs, *Theor. Chem. Acc.* **1976**, *42*, 33-45; b) R. Ahlrichs, C. Ehrhardt, *Chem. Unserer Zeit.* **1985**, *19*, 120-124.

[10] T. Lu, F. Chen, *J. Comput. Chem.* **2012**, *33*, 580-592.

[11] P. Plessow, *J. Chem. Theory Comput.* **2013**, *9*, 1305–1310.

[12 O. Treutler, R. Ahlrichs, *J. Chem. Phys.* **1995**, *102*, 346-354.

[13] F. Weigend, *Phys. Chem. Chem. Phys.* **2006**, *8*, 1057-1065.

[15] D.A. McQuarrie, J.D. Simon, *Molecular Thermodynamics* ISBN 978-1891389054 (University Science Books, **1999**)

[16] C. Lee, W. Yang, R. G. Parr, *Phys. Rev. B.* **1988**, *37,* 785–789.

[16] F. Weigend, R. Ahlrichs, *Phys. Chem. Chem. Phys*. **2005**, *7*, 3297-3305.

[17] A. Schäfer, A. Klamt, D. Sattel, J. C. W. Lohrenz, F. Eckert, *Phys. Chem. Chem. Phys.* **2000**, *2*, 2187-2193.

[18] S. Grimme, J. Antony, S. Ehrlich, H. Krieg, *J. Chem. Phys.* **2010**, *132*, 154104.
